# What Makes Parents of Young Children Stressed? A Systematic Review of Quantitative Studies

**DOI:** 10.1007/s10567-026-00573-7

**Published:** 2026-06-02

**Authors:** Dora d’Orsi, Carolina Garraio, Rita Santos, Marisa Matias, Manuela Veríssimo, Eva Diniz

**Affiliations:** 1 William James Center for Research, Ispa—Instituto Universitário, Rua Jardim do Tabaco, 34, 1149-041 Lisbon, Portugal; 2 Center for Psychology at University of Porto, Faculty of Psychology and Education Sciences, University of Porto, Rua Alfredo Allen, 4200-135 Porto, Portugal; 3 Ispa—Instituto Universitário, Rua Jardim do Tabaco, 34, 1149-041 Lisbon, Portugal

**Keywords:** Parental stress, Early childhood, Systematic review, Parenting, Quantitative studies

## Abstract

The arrival of a baby and the early years of a child’s life represent a critical period for parents, often marked by increased parental stress that can impact both their well-being and the child’s development. Although parental stress has been widely studied, no systematic review has yet focused specifically on early childhood. To address this gap, we conducted a systematic review synthesizing evidence on factors examined as antecedents of parental stress during the first three years postpartum. Following PRISMA guidelines, we included 108 quantitative studies published in the past 12 years that investigated variables statistically modeled as predictors of parental stress. Key determinants of parental stress were identified at three levels: (1) at the personal level, internalizing symptoms, adverse childhood experiences, and perinatal negative experiences were related to higher stress; (2) at the relational level, marital satisfaction and coparenting quality were associated with lower stress; and (3) at the contextual level, social support from friends and family served as a protective factor of parental stress, while children’s developmental problems served as risk factors. Despite the increasing number of longitudinal studies and the growing inclusion of fathers in research, few studies have focused on participants from social minority groups or from non-Western contexts. These findings may contribute to the development of effective strategies to support families during the early years of parenthood.

**Supplementary Information:**

The online version contains supplementary material available at https://doi.org/10.1007/s10567-026-00573-7.

## Introduction

Parental stress can be defined as a specific type of stress that occurs when parents perceive the demands of child-rearing as exceeding their available resources to cope with them (Abidin et al., 2022; Deater-Deckard & Panneton, 2018). It is a prevalent phenomenon among parents worldwide (Adams et al., 2021; Buddelmeyer et al., 2018), with recent data indicating that 33% of parents report high levels of stress (American Psychological Association, 2024). The early years of a child’s life, particularly the 0–3 age range, are a critical period for parental stress (World Health Organization, 2022), as parents must manage multiple activities, such as develop or adapt their parenting skills, adapt their daily routines to meet the baby's needs, and renegotiate their roles within the family and in the labor market (Kline et al., 1991; Kuersten-Hogan & McHale, 2021). Additionally, infants and toddlers are highly dependent on their caregivers, making the care of young children a physically, emotionally, and practically demanding task, which can be a potential source of stress for many parents (Da Costa et al., 2021; Gillis et al., 2019).

The experience of (parental) stress, when well handled, can be an important adaptive response, particularly during life transitions (Lazarus & Folkman, 1984; Yim et al., 2015). However, when parental stress is frequently high, it can lead to a wide range of consequences: for parents, such as greater depression, anxiety, and parental burnout (Chen et al., 2022; Johansson et al., 2021; Mikolajczak et al., 2018); for parenting quality, such as lower-quality of parenting behaviors (Camisasca et al., 2022; Huang, 2021); and for child development, such as increased emotional and behavioral problems in children (Cho et al., 2023; Kelly et al., 2022). Moreover, parental stress has broader social consequences. When parents experience elevated stress, they may perform less effectively at work and increase the demand for medical and mental health services, resulting in increased costs for employers and healthcare systems (American Psychological Association, 2024). Understanding the determinants that increase or reduce parental stress in early childhood is crucial for intervening with parents and mitigating its negative consequences at the individual, familial, and societal levels.

Although research on parental stress has expanded, findings are dispersed across disciplines, vary in methodological quality, and often focus on isolated determinants (see Fang et al., 2022, for a previous review), which limits the ability to draw clear conclusions about which factors contribute to or buffer against parental stress. A systematic review is therefore needed to consolidate existing evidence, identify possible predictors, and inform targeted intervention and policy efforts. Thus, this systematic review aims to synthesize evidence on factors examined as antecedents of parental stress in the first three years after birth.

### Parental Stress Models

Parental stress describes the specific stress experienced by parents when the demands of parenting (stemming both from the child and other life domains) exceed their perceived coping resources (Abidin et al., 2022; Deater-Deckard & Panneton, 2018). It is a multidimensional construct that can be understood at different levels (Deater-Deckard, 2004). From a micro perspective, it refers to feeling overwhelmed by childcare itself (Cronin et al., 2015). From a broader, contextual perspective, it involves balancing childcare with other responsibilities, such as paid work, maintaining a couple relationship, sustaining friendships, and managing household tasks (Cronin et al., 2015).

The variety of models developed to study parental stress reflects this multidimensional nature. From a micro perspective, Abidin’s Parenting Stress Model (2006) suggests that parental stress arises from a combination of parents’ internal characteristics (e.g., personality, low sense of competence), children’s characteristics (e.g., difficult behavior, health problems), and dysfunctional parenting behaviors. Even though this model has made a substantial contribution to the field, it has been criticized for underestimating the influence of contextual factors (e.g., cultural context) in which parenting takes place (Cronin et al., 2015; Louie et al., 2017).

From a broader perspective, ecological models can provide a more comprehensive framework for understanding the dynamism and complexity of parental stress. For example, the Double ABC-X model of family stress and coping (McCubbin & Patterson, 2014; Rosino, 2016) conceptualizes stress as the result of an interaction between stressors (A), family resources (B), and the family’s appraisal of the situation (C), which together determine whether the outcome is adaptation or crisis (X). These resources may operate at multiple levels (within individual family members, the family unit, or broader community and societal systems), highlighting the interplay between personal and contextual factors (Rosino, 2016). Similarly, the Family Stress Model (Masarik & Conger, 2017) underscores how external stressors, such as economic hardship or natural disasters, influence children’s adjustment indirectly through parents’ mental health, coparenting dynamics, and parenting behaviors. Additionally, other ecological models widely used in parenting research (i.e., Belsky, 1984; Bronfenbrenner & Morris, 1998; Cabrera et al., 2014), though not specifically developed to address parental stress, can serve as a valuable complement to understand its determinants. These models conceptualize parenting as a dynamic, reciprocal process shaped by personal factors, relational processes, and broader social and ecological contexts (Cabrera et al., 2014).

Taken together, the models described above highlight the importance of examining parental stress within a personal and contextualized, multilevel framework. Accordingly, in this systematic review, we aimed to identify and synthesize variables examined as predictors of parental stress during early childhood through a multidimensional lens. Hence, we considered determinants operating at three levels: (1) the personal level, encompassing factors within parents as individuals, such as sociodemographic characteristics, personal history, physical and mental health, beliefs, and emotional experiences; (2) the relational level; referring to parents’ immediate dyadic relationships within the family system, such as parent–child relationship and mother-father relationship; and (3) the contextual level, which includes influences from the immediate context (e.g., family configuration, child characteristics, social network) and the broader sociocultural environment (e.g., work conditions, and prevailing social, political, and economic circumstances) in which parents are embedded.

### Determinants of Parental Stress

Parental stress was previously examined in relation to diverse personal, relational, and contextual determinants. For instance, personal factors such as parental feelings of incompetence (Hildingsson et al., 2014; Seah & Morawska, 2016), being from an ethnic minority (Chung et al., 2013; Franco et al., 2010), presenting psychopathological symptoms (Seah & Morawska, 2016; Van Der Oord et al., 2006), or having pregnancy-related problems (Yu et al., 2011) were associated with higher parental stress. Relational aspects within the family also seem to influence parental stress, such as having a partner with high stress levels (Ngai & Ngu, 2014). In contrast, perceived higher marital quality (deMontigny et al., 2020; Dong et al., 2022; Pinquart, 2018) and a positive coparenting relationship (deMontigny et al., 2020; Fagan & Lee, 2014; Nomaguchi et al., 2017; Zhang et al., 2021) were related to lower parental stress. Finally, at a contextual level, work-related difficulties (Coba-Rodriguez & Lleras, 2022; Hwang & Jung, 2020) were associated with higher stress levels, whereas greater social support from family and friends was associated with lower parental stress (Barnett et al., 2013; Zhang et al., 2021). Child-related factors can also contribute to increasing parental stress, such as child behavior problems (Pinquart, 2018). Within the contextual level, the global COVID-19 pandemic in recent years forced many parents into lockdown with their children for weeks or even months. This context, marked by childcare closures, remote work while caregiving, job loss or reduced income, fear of infection, and loss of social support, significantly heightened parental stress (Adams et al., 2021; Aldoney et al., 2022; Chen et al., 2022).

Many of these findings have been presented in previous systematic reviews and meta-analyses. However, most of these reviews focused on parents of children with developmental challenges or chronic conditions (e.g., Barroso et al., 2018; Pinquart, 2018; Schappin et al., 2013). This emphasis has contributed to a gap in the understanding of parental stress among parents of typically developing young children. Additionally, although some reviews examined typical parenting during the perinatal period, they tend to focus on general stress rather than on parental stress (Philpott et al., 2017; Saur & Dos Santos, 2021). A recent systematic review explored determinants of parental stress in the context of typical development (Fang et al., 2022), but its scope was limited to studies using the Parenting Stress Index (PSI; Abidin et al., 2006) and included parents of children up to 12 years of age. While the PSI is often regarded as the gold standard for measuring parenting stress, it is not the only relevant instrument (Øygarden et al., 2022). Moreover, as previously discussed, the first three years following a child’s birth involve unique challenges, meaning the sources of stress during this period may differ significantly from those in later stages of parenthood (Kline et al., 1991). Hence, as addressed by Fang and colleagues (2022), “future (review) studies could focus on a smaller age group to study in depth the factors associated with parenting stress. In such studies, it would be feasible to include several instruments to assess parenting stress (…)” (p. 16).

Thus, the aims of the present study are to: (1) characterize the included studies in terms of their target populations and methodological approaches, (2) systematize the existing quantitative evidence on factors examined as antecedents of parental stress, focusing on the 0–3 years period, and (3) identify gaps in the literature, suggesting directions for future studies to build a more comprehensive framework for examining parental stress determinants.

## Method

### Registration

The systematic review protocol was registered in the International Prospective Register of Systematic Reviews (PROSPERO) on October 14, 2023 and updated on April 23, 2025, with the registration number CRD42023464874. The PRISMA 2020 guidelines for systematic reviews were used to guide all steps (article identification, screening, extraction, and reporting) of this review (Page et al., 2021).

### Search Strategy

A systematic literature search was conducted in seven electronic databases (MEDLINE, APA PsycInfo, Psychology and Behavioral Sciences Collection, APA PsycArticles, Scopus, Web of Science, and PubMed). These databases were chosen due to their broad coverage across psychology, health sciences, social sciences, and related multidisciplinary fields. The search strategy included the Boolean terms “OR”/ “AND” and truncation “*”. Keywords and their synonyms were combined: (parent* stress OR maternal stress OR parent* hassles) AND (infant OR infancy OR toddler OR child* OR baby OR babies) for search in title, abstract, and keywords. The search process was independently carried out by two researchers to enhance the reliability of the results. Results from our search showed an increase in publications from 2013 onwards. Therefore, we chose to focus our review on studies published between January 1, 2013, and May 16, 2025, to capture the recent portrait of research on parental stress.

### Inclusion and Exclusion Criteria

For abstract screening, we established the following criteria: (1) empirical articles with available abstract published in peer-reviewed journals; (2) articles published in English, Portuguese, or Spanish, the languages spoken by the authors; (3) quantitative studies in which parental stress was conceptualized and statistically modeled as an outcome variable; (4) studies focused on adult parents (i.e., > 18 years old) of children aged 0–3 years; (5) studies that focused on parents without diagnosed physical and/or mental illness, or addictive substance usage, and on children without health or developmental conditions (e.g., prematurity, autism); (6) studies not conducted with families at risk (e.g., parental incarceration); (7) studies not designed to develop, adapt, or validate an instrument or an intervention.

The following exclusion criteria were defined for full-text screening: (1) studies in which parental stress was treated as mediator; (2) studies in which data was examined as part of cluster analyses, which did not allow to retrieve conclusions regarding parental stress determinants; (3) studies that did not use a specific psychological measure for parental stress, or that used physiological measures of stress (since physiological measures of stress do not allow us to distinguish general stress from parental stress); and (4) studies in which parents had participated in any psychosocial intervention related to parenting or wellbeing (to avoid potential bias in parental stress outcomes). After full-text screening, we conducted a quality assessment of the included studies (see the Sect. "Quality appraisal and data extraction" below). Given the large number of studies meeting the inclusion criteria, we adopted a more conservative approach and introduced an additional exclusion criterion: (5) studies rated as low quality (below 70%; Kmet et al., 2004).

For the purpose of this review, we extracted only the quantitative data of mixed-methods studies. Also, if the study compared the general population with specific samples (e.g., neurotypical vs. children with ADHD), we only retrieved data from the general population group. For longitudinal designs, we only considered data collected within the first three years of the child’s life. In studies that used cross-sectional designs, we focused exclusively on factors that were theoretically framed and statistically tested as predictors of parental stress; the directionality, therefore, reflects conceptual and analytical modeling rather than causal inference.

### Study Selection

**Fig. 1:**
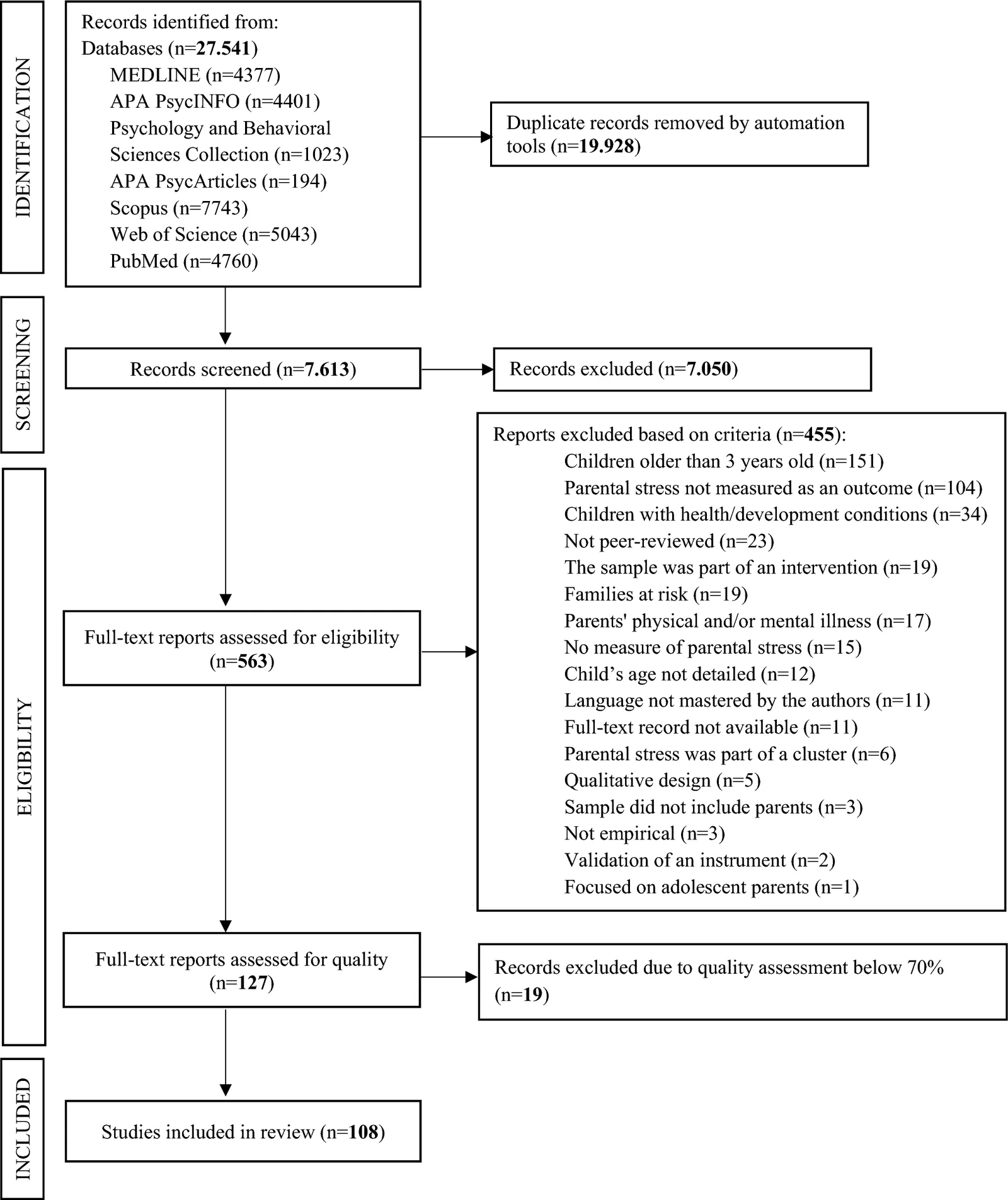
Flowchart of the study identification and selection process

We identified 27,541 records through the search. After removing duplicates, we retained 7,613 records. Two reviewers (first and third authors) independently screened titles and abstracts and excluded 7,050 records at this stage. The remaining 563 full texts were then screened independently by two reviewers. The reviewers resolved any disagreements by consensus, resulting in 127 studies that met all the inclusion criteria. We then assessed the quality of the studies and rated 19 studies as low quality, which we excluded, leaving 108 studies for this review (Fig. 1). We exported and managed all references using the Rayyan software.

### Quality Appraisal and Data Extraction

After the full-text screening and before data extraction, we assessed the quality of the included studies using the Standard Quality Assessment Criteria for Evaluating Primary Research Papers from various fields (QualSyst) for quantitative studies (Kmet et al., 2004). The QualSyst is a 14-item tool designed for methodological and bias assessment in studies with various designs. QualSyst items comprise the study’s objective (e.g., “Question or objective sufficiently described”), design (e.g., “Design evident and appropriate to answer study question?”), sample selection, characterization, and size (e.g., “Sample size appropriate?”), type of outcome measure (e.g., “Outcome measure(s) well defined and robust to measurement/misclassification bias?”), analysis (e.g., “Analysis described and appropriate?”, “Controlled for confounding?”), and results (e.g., “Do the results support the conclusions?”). Due to the nature of the included studies, we excluded three items: random allocation (item 5), blinding of investigators (item 6), and blinding of subjects (item 7). We scored each item based on whether the study met the criterion (0 = no, 1 = partially, 2 = yes). The scores from the remaining 11 items were summed to produce a total score, which was then converted into a percentage (total sum score divided by the maximum possible score of 22). Studies were rated as “excellent” (> 80%), “adequate” (70%–79%), or “low” (< 70%; Kmet et al., 2004). All items and criteria used for article evaluation can be found in the Supplementary Materials. As mentioned in the “Inclusion and exclusion criteria” section, we rated 19 articles as low quality (< 70%) and excluded them from further analysis. Among the remaining studies (*n* = 108), we rated most (*n* = 86) as excellent and 23 as adequate.

After the quality appraisal, we analyzed and extracted the data using a categorization system developed for this study, to characterize the studies for: general characteristics (country of origin, design; type and origin of data); study’s aim; general characteristics of participants (i.e., mother or father, age, relationship status, employment status, educational level, income, ethnicity, and primiparity) and their children (i.e., age, sex); statistical analysis, measure of parental stress and predictor variables, control variables, and results.

Given the large number of studies included, we began the quality assessment and the data extraction processes with a training phase using 20% of the articles. At that stage, two researchers independently extracted data from these 20% studies, and any discrepancies were discussed until a consensus was reached. After that, once consistency in the evaluation and extraction processes was established, the remaining 80% of the studies were divided among four authors, who evaluated and extracted them individually using the predefined spreadsheet.

## Results

### Characteristics of the Included Studies

Table 1 summarizes the characteristics of the studies included in this review. The studies were predominantly conducted in the North America (37 studies in the U.S. and five in Canada) and in European countries (nine in Italy, nine in Sweden, five in Germany, four in the Netherlands, four in the UK, three in Norway, two in Ireland, two in Portugal, and one each in Belgium, Finland, Scotland, and Spain). Twelve studies were conducted in Asia (five in Japan, four in China, and three in South Korea), followed by the Middle East (six in Israel), Oceania (three in Australia), and South America (one in Chile). Two studies included samples from multiple countries: one from Finland and the UK, and another from the UK, the Netherlands, and France.

**Table 1:** Characteristics of the studies included in the systematic review (n = 108)

	*n*	%		*n*	%
*Geographical region*			*Assessment of parental stress*		
North America	42	38.9	Self-report	96	88.9
Europe	42	38.9	Self-report and other measurements	12	11.1
Asia	12	11.1	*Who reported*		
Middle east	6	5.6	Mother	62	57.4
Oceania	3	2.8	Father	8	7.4
Samples from multiple countries	2	1.8	Both	38	35.2
South America	1	0.9	*Children’s age range*		
*Study design*			Infant (0–12 months)	51	47.2
Longitudinal	65	60.2	Toddler (13–35 months)	36	33.3
Cross-sectional	43	39.8	Mixed ages until 36 months	21	19.4
*Type of data*			*Child gender*		
Original sample	84	78.8	Both boys & girls	50	46.3
Secondary data	24	22.2	Not mentioned	58	53.7
*Measure of PS*			*First child?*		
Parenting Stress Index	64	59.2	Mostly first	33	30.6
Items adapted from PSI (FFCWS)	16	14.8	No	51	47.2
Parental Stress Scale	11	10.2	Not mentioned	24	22.2
Swedish Parenthood Stress Questionnaire	9	8.3			
Other	8	7.4			

In terms of study design, 60.2% of studies had a longitudinal design, and most studies relied on original samples (78.8%). A relevant proportion of studies utilizing secondary data (*n* = 21) were conducted in the United States, mainly (*n* = 17) drawing on the Future of Families & Child Wellbeing Study (FFCWS). The remaining studies relying on secondary data were conducted in Ireland (*n* = 2) and in Scotland (*n* = 1).

Most studies (74%) measured parental stress with the PSI (Abidin et al., 2006), followed by 10.2% that used the Parental Stress Scale (Berry & Jones, 1995). Parental stress was exclusively self-reported; none used multi-informant or other-report measures. A small proportion (11.1%) also incorporated additional methods (e.g., observation), but these were used to measure variables other than parental stress (e.g., maternal mind-mindedness). Regarding informants, 57.4% of the studies included only mothers, 35.2% included both parents, and 7.4% included only fathers. Among studies that involved both mothers and fathers (*n* = 38), researchers often analyzed them collectively as “parents” (*n* = 20), rarely examining differences between mothers and fathers or interdependence within parental dyads. Nearly half of the studies (47.2%) focused on infants (0–12 months) and on families with more than one child (47.2%). Regarding the sex of the focal child (i.e., the infant/toddler aged 0–3 years), about 46.3% of the studies included an equal distribution of boys and girls.

### Participants’ Characteristics

Table 2 outlines participants’ characteristics. Sample sizes varied widely across studies, but most included more than 100 parents, typically mothers. In most studies, parents’ mean age ranged from 31 to 35 years (in 43.6% of studies including mothers and 61.7% including fathers). Participants were predominantly Caucasian (in 42.6% of studies including mothers and 40.4% including fathers), likely reflecting the demographics of the countries where the studies took place. Most samples included parents who were married or cohabiting (66%). Nearly half of the studies did not report participants’ employment status, but those that reported typically (28.7% of mothers and 38.3% of fathers) focused on employed parents. In terms of education, most studies included parents with high educational levels (in 53.5% of studies including mothers and 36.2% including fathers), although several included participants with diverse educational backgrounds. Regarding socioeconomic status (SES), studies were fairly balanced across low, middle, and high SES groups, as well as samples including multiple SES levels. However, some studies (13%) did not provide information on participants’ SES.

**Table 2:** Sample characteristics of studies included in the systematic review (n = 108)

	Mothers (*n* = 101)		Fathers (*n* = 47)	
	*n*	*%*	*n*	*%*
*Sample size*				
< 100	20	19.8%	15	31.9%
100–300	21	20.8%	10	21.3%
301–1000	26	25.7%	11	23.4%
> 1000	34	33.7%	11	23.4%
*Sample’s mean age (years)*				
18–25	1	1.0%	0	0.0%
25–30	39	38.6%	6	12.8%
31–35	44	43.6%	29	61.7%
> 35	4	4.0%	9	19.1%
Mean age not mentioned	8	8.0%	3	6.4%
*Ethnic background*				
Caucasian	43	42.6%	19	40.4%
Black	1	1.0%	0	0.0%
Asian	9	8.9%	2	4.3%
Latino	3	3.0%	1	2.1%
Middle East	6	5.9%	4	8.5%
Sample including multiple ethnic groups	19	18.8%	5	10.6%
Not mentioned	19	18.8%	16	34.0%
*Relationship status*				
Mostly married/cohabiting	67	66.3%	31	66.0%
Mostly divorced/separated	2	2.0%	0	0.0%
Sample including married and divorced parents	11	10.9%	2	4.3%
Not mentioned	21	20.8%	14	29.8%
*Employment status*				
Most employed	29	28.7%	18	38.3%
Most unemployed/parental leave	6	5.9%	0	0.0%
Sample including different employment statuses	15	14.9%	4	8.5%
Not mentioned	50	49.5%	25	53.2%
*Education*				
High (> high school)	54	53.5%	17	36.2%
Medium (high school)	9	8.9%	3	6.4%
Low (< high school)	4	4.0%	1	2.1%
Sample with diverse education levels	27	26.7%	18	38.3%
Not mentioned	7	6.9%	8	17.0%
*Socioeconomic status*				
Low	15	14.9%	4	8.5%
Middle	14	13.9%	8	17.0%
High	19	18.8%	7	14.9%
Sample including multiple SES groups	13	12.9%	6	12.8%
Not mentioned	40	39.6%	22	46.8%

### Factors Related to Parental Stress

Inspired by the Parenting Stress Model and by ecological parenting models, we categorized the variables related to parental stress into three categories: (1) Personal aspects (*n* = 75), examining parent-related variables, such as personal history, sociodemographic characteristics, physical and mental health, and parents experiences on the perinatal phase; (2) Relational aspects (*n* = 35), including immediate relationships within the family, such as the mother-father relationship and the parent–child relationship; and (3) Contextual aspects (*n* = 57), such as extra-familial context, child’s characteristics, and sociocultural aspects. These three levels will be detailed next. The studies corresponding to each level can also be found in the tables provided in the Supplementary Material. Importantly, all factors presented derive from studies (cross-sectional or longitudinal) in which parental stress was theoretically framed and statistically modeled as the outcome variable, while the factors outlined were treated as predictors. To facilitate reporting of the 108 included studies, each study was assigned a unique identifier (S1–S108) for use in the Results section and Supplementary Tables; full citations for all included studies are presented in Appendix A.

#### Personal Aspects Related to Parental Stress

This category was the most extensively studied (*n* = 75) and includes parents’ personal aspects that are related to parental stress, such as parents’ personal history, sociodemographic characteristics, general health, psychological aspects, and parents’ experiences in the perinatal phase.

##### Parents' Personal History

 This sub-category (*n* = 13) covers factors related to mothers’ and fathers’ personal histories before becoming parents, focusing on variables such as adverse childhood experiences (ACEs), attachment, previous parenting experiences, and role models. Among mothers, ACEs were consistently associated with higher levels of parental stress (*n* = 7; S3, S50, S67, S79, S89, S90, S100). Two studies with mothers found indirect pathways from more ACEs to higher maternal stress: one through increased depressive symptoms (S79) and the other through lower maternal perceptions of social support (S89). For fathers, the findings were mixed and varied across study designs. In the only longitudinal study with fathers (S74), ACEs did not predict paternal stress directly; instead, effects operated indirectly via fathers’ prenatal depression, anxiety, and spousal disharmony. In contrast, two fathers-only cross-sectional studies reported a positive direct association between cumulative childhood trauma and parental stress (S67, S89). One of these cross-sectional studies also found that cumulative childhood trauma was indirectly related to paternal stress through lower social support (S89).

Other relevant aspects of parents’ personal histories were their attachment quality and how they perceived the care they received growing up. Insecure attachment style was related to greater parental stress in studies conducted across Europe and Japan, although this variable was examined more frequently in mothers (S56, S59, S61, S98) than in fathers (S61). A retrospective study with mothers found that receiving high-quality rearing from their own fathers during childhood was associated with lower maternal stress in adulthood, an effect mediated by mothers’ greater current relationship satisfaction with their fathers (S79). In the same study (S79), mothers who had received high-quality rearing from their own mothers during childhood reported lower maternal stress in adulthood, an association explained by the internalization of maternal care regulation. Finally, another study showed that having previous parenting experience was related to more stress in mothers (S83), but not in fathers (S83), whereas having a parental role model was related to lower stress for both parents (S83).

##### Sociodemographic Aspects

 The relation between parents’ sociodemographic characteristics (e.g., age, education, income) and parental stress was frequently examined across studies (*n* = 43). Education was the most assessed variable in this sub-category (*n* = 22), with mixed findings. A group of studies (eight focusing on mothers, S24, S50, S62, S65, S69, S83, S86, S95, and two on fathers, S49, S62), often using longitudinal secondary data, found a negative association between educational level and parental stress. In contrast, a similar number of studies (*n* = 9; S13, S29, S39, S67, S76, S77, S89, S101, S106), six of which were cross-sectional, reported that parents with higher education experienced greater stress. Specifically, one study in the U.S. (S101) found that higher education was related to greater stress only among White mothers. However, five studies (S74, S83, S90, S103, S108) found no significant association between education and parental stress across diverse samples and study designs. One study in Sweden (S83) reported that lower education was related to higher stress for mothers, but not for fathers.

Most studies found that higher household income was associated with lower parental stress in both mothers and fathers (*n* = 11; S6, S13, S20, S24, S29, S49, S64, S76, S95, S98, S105). Studies reporting null associations were mostly conducted in highly developed countries, such as Norway (S74) and Germany (S103), as well as in one diverse U.S. sample (S46) and in one study during the COVID-19 pandemic (S108). A single study in Israel (S90) reported the opposite pattern, with higher income related to higher stress in mothers. One Australian study found a null association for mothers but a positive association for fathers (S72). Additionally, a U.S. study during the COVID-19 pandemic (S88) showed that, before the pandemic, higher income was related to lower stress, but parents with lower household income experienced a greater increase in stress over the course of the pandemic.

Concerning age, most studies (*n* = 7, S39, S67, S74, S89, S90, S103, S108) did not find an association between parental age and stress. However, some studies reported a positive association between parental stress and mothers’ age (*n* = 5; S1, S31, S67, S86, S106), while one study observed a negative correlation between parental stress and fathers’ age (S89).

Having a partner (married or cohabiting) was associated with lower parental stress in five studies with mothers (S1, S50, S63, S95, S107). Among these, three included low-income samples (S50, S63, S95), while the other two were conducted during the COVID-19 pandemic in Chile and the U.S. (S1, S107). In contrast, three European studies reported no significant association (S39, S103, S108). One American study with a low-income sample found that re-partnered fathers reported lower stress, whereas mothers did not (S62). A secondary data analysis study showed that mothers with children from different partners did not report greater stress (S27).

Being employed was associated with lower parental stress for both mothers and fathers (S16, S49, S50, S62), among low-income (S49, S50, S62) and middle-class parents (S16). A study conducted in Ireland found a negative association for mothers only, with no significant effect for fathers (S55).

Migrant background was generally associated with higher parental stress, as reported in eight studies with mothers (S1, S24, S39, S50, S76, S86, S95, S101) and two with fathers (S39, S62). One U.S. study found that Latino fathers experienced greater stress than White fathers, while no association was observed for mothers (S62). In contrast, another U.S. study reported lower stress among Black and Hispanic fathers compared to White fathers (S49); however, this association only emerged after controlling for sociodemographic variables. That is, Black and Hispanic fathers reported lower levels of stress than White fathers only when they had equal amounts of human capital (S49).

Findings on the number of children were mixed. Three studies from Northern Europe (Sweden, Germany, and Norway) with highly educated, high-SES samples found that having previous children was associated with lower stress among mothers (S50) and fathers (S37, S74). In contrast, six studies reported that having more children was related to higher parental stress (S1, S29, S41, S49, S98, S103). In the U.S., one study observed this positive association for mothers but not for fathers (S62). Two studies found no association (S13, S107).

Finally, studies examining family type focused on samples including both parents and compared heterosexual biological parents with homosexual or adoptive parents. One study found no differences in parental stress between partners in gay, lesbian, and heterosexual couples (S80), while another reported lower stress in lesbian couples in comparison to heterosexual parents (S12). One study found that adoptive parents reported higher parental stress than biological parents (S8), while another found no association (S15).

##### General Health

This sub-category covers parents’ general health variables that predict parental stress, such as physical health and sleep quality. It was the least studied sub-category (*n* = 8), but findings were consistent. Parental sleep difficulties were examined only in European mothers and were positively and directly correlated with parental stress (S14, S55, S93). One study found an indirect effect from sleep difficulties to maternal stress through increased depressive symptoms (S14). In contrast, good physical health was associated with lower parental stress for both mothers and fathers (S4, S7, S55, S77, S106).

##### Psychological Aspects

This sub-category includes parents’ emotional and cognitive factors associated with parental stress (*n* = 39). Internalizing symptoms were the most extensively studied variable (*n* = 27), including depressive symptoms, poor mental health, psychological history, prenatal depression, anxiety, dysregulation, and trait anger. Overall, internalizing symptoms were consistently associated with higher parental stress in mothers (*n* = 22; S9, S14, S17, S23, S24, S33, S44, S47, S60, S62, S64, S67, S75, S78, S86, S88, S96, S101, S102, S104, S106, S107) and fathers (*n* = 8; S23, S28, S44, S47, S71, S72, S75, S82). One study with mothers (S72) found no association between a history of anxiety or depression and parental stress. Anxiety and depressive symptoms were indirectly associated with maternal stress through attachment patterns (S102) and indirectly associated with paternal stress through spousal disharmony (S74). Also, in both parents, problematic video game use was associated with increased depressive symptoms, which, in turn, was associated with higher parental stress (S75).

Studies conducted during the COVID-19 pandemic reported that worry about infection was related to higher parental stress in mothers and fathers (S29, S107, S108), although one study found no association (S104). In addition, COVID-19-related stress increased maternal stress indirectly through lower maternal resilience (S107).

Finally, we grouped studies that examined parents’ cognitive aspects related to their parenting role, such as self-efficacy, meaning in life, and resilience. For mothers, acting with awareness (S11), effortful control (S64), sense of coherence (S47), mind-mindedness (S91), and resilience (S107) were related to lower parental stress, whereas identity impairment (S67) was related to higher stress. Greater embodied mentalizing was indirectly related to lower parental stress through improved coparenting quality (S73). For fathers, sense of coherence (S47), positive attitudes about fatherhood (S49), and sense-making (S19) were associated with lower parental stress. In samples including both parents, the use of prementalizing mode to reflect on the child (S61) and meaning in life (S77) were related to lower parental stress. However, self-efficacy revealed mixed findings: five studies found that greater self-efficacy was related to lower parental stress, in mothers (S76, S83) and fathers (S71, S72, S83), whereas one cross-sectional study of Chinese mothers (S87) found no association. Null associations were reported for other cognitive aspects, e.g., reflective functioning (S19, S67, S73).

##### Perinatal Factors

This sub-category covers parents’ experiences during pregnancy, delivery, and the postpartum period (*n* = 25). Intended pregnancy (S5, 57) and pregnancy risk (S23) were associated with lower postnatal stress. In contrast, mothers who had considered abortion or had been through a perinatal loss reported higher postnatal stress (S18, S26). Regarding prenatal emotional experiences, positive prenatal feelings were related to lower postnatal stress in both parents (S37), while prenatal anxiety was related to higher stress in mothers (S85, S96) and fathers (S74). Pregnancy-related concerns (e.g., fear of childbirth) were associated with higher stress for mothers (S23) and fathers (S38). COVID-19 pandemic-related concerns about pregnancy and childbirth were not related to maternal stress (S104). Parents who conceived through egg donation, IVF, or donor insemination did not experience higher stress levels compared to those who conceived spontaneously (S10, S43).

Regarding delivery, a negative birth experience was associated with higher parental stress for both parents (S20, S39, S60, S77, S91, S92, S93). Postpartum traumatic stress in mothers was also related to higher maternal stress through lower mindfulness (S91) and exacerbated by sleep disturbances (S93). Rapid labor was related to increased postpartum stress for mothers, but not fathers (S55), and cesarean delivery was associated with higher stress in Japanese mothers (S54).

Breastfeeding stress was associated with higher maternal stress (S76). However, mothers in mixed feeding (i.e., breastfeeding combined with formula) showed greater stress than exclusive breastfeeding or formula feeding (S53). The use of pacifiers was associated with lower maternal stress in one UK study (S99).

#### Relational Factors

This category encompasses mothers’ and fathers’ relationships within the family, namely the mother-father relationship (including cross-over effects between parents) and the parent–child relationship (*n* = 35).

##### Mother-Father Relationship

We grouped variables into two aspects: marital relationship (*n* = 18) and coparenting relationship (*n* = 11). Marital relationship quality covered diverse variables, including marital satisfaction, spousal disharmony, attachment to the partner, and couple leisure. Most studies (*n* = 14, S2, S17, S19, S22, S25, S27, S32, S46, S55, S56, S77, S83, S94, S107) reported that higher relationship-quality indicators (e.g., marital intimacy, dyadic adjustment) were associated with lower parental stress. Shared couple leisure was related to lower parental stress indirectly through improved coparenting quality for mothers (S94) and fathers (S107). Similarly, one study reported a positive association between relationship anxiety and maternal stress (S56), and another found a positive link between spousal disharmony and paternal stress (S74). Four studies with mothers (S3, S56, S84, S94) and four with fathers (S3, S19, S84, S94) reported null associations, which were typically observed in studies with small samples (S3, S19, S56) or relying on secondary data (S84, S94).

Most studies reported that higher coparenting quality was associated with lower parental stress for mothers (*n* = 7; S22, S24, S45, S63, S69, S73, S94) and fathers (*n* = 2; S49, S94). Conflictual coparenting was related to greater paternal stress (S25), and family conflict was related to greater stress in both parents (S29).

##### Cross-Over Effects

This sub-category covers cross-over effects between parents, i.e., when one parent’s characteristics influence the other parent’s stress (*n* = 14). Studies focused on two main aspects: partner psychological factors (e.g., dyadic coping; *n* = 6) and partner involvement with the child (*n* = 7) as predictors of the other parent’s stress.

Regarding partner psychological factors, fathers’ dyadic coping (S2, S3) and fathers’ changes in identity (S19) were related to lower maternal stress. Similarly, mothers’ perceived dyadic coping (S2) was related to lower paternal stress. Fathers’ avoidant attachment (S67) and fathers’ parental stress (S15, S47) were associated with higher maternal stress, whereas mothers’ sense-making (S19), relatedness (S67), and parental stress (S15, S47) were associated with higher paternal stress. Four studies reported null associations (S3, S15, S19, S67).

Regarding partner involvement with the child, four studies found that greater father involvement was associated with lower maternal stress (S22, S58, S63, S87), whereas its absence was associated with higher maternal stress (S27). One study found that mothers’ positive involvement was related to lower paternal stress (S49). One study with mostly divorced mothers reported no association between fathers’ involvement and maternal stress (S86).

##### Parent–Child Relationship

Aspects related to the quality of parent–child relationships were not extensively studied (*n* = 9). Still, findings revealed that parental bonding (S21, S28), parent–child shared emotions (S52), and parental responsiveness (S72) were associated with lower parental stress in mothers and fathers. A longitudinal study found that higher prenatal bonding was related to lower parental stress through higher postnatal bonding at 6 months (S21). Secure attachment to the child was negatively related to mothers’ stress, but not to fathers’ (S72). Greater involvement with the child was associated with lower parental stress for mothers and fathers (S9, S49, S58, S86). An exception was a cross-sectional study mainly with ethnic minorities, in which a higher frequency of engagement was associated with higher maternal stress (S62).

#### Contextual Factors

This category includes factors related to the context of parenting, such as the extra-familial context, children’s characteristics, and sociocultural factors (*n* = 57).

##### Extra-Familial Context

This category includes aspects related to the broader environment in which the family is embedded, such as social networks, external support, and parents' work-related aspects. Support from friends and family was extensively studied (*n* = 19), mostly revealing an inverse association with parental stress for both parents (S6, S14, S27, S33, S50, S65, S69, S70, S76, S86, S87, S89, S98, S100, S101, S107). However, this association was not found during COVID-19 (S104) and in a sample of Mexican-origin mothers in the U.S. (S34).

Sources of external support, such as neighborhood facilities (S41, S48, S68, S97), were related to lower maternal stress. Guidance from health care professionals (S41, S95) was associated with lower parental stress, whereas support from online communities showed no association (S36). In one study with Chinese parents, access to childcare services was associated with lower parental stress (S41). Also, in a study of Chinese dual-earner parents (S40), there was no difference in stress levels between those who relied on parental childcare and those who used non-parental childcare (e.g., daycare, babysitters). Restrictions of family support services during the COVID-19 pandemic were related to increased parental stress in both parents (S29), as well as having a child in daycare during this period (S103).

Work-related factors were less explored and mainly studied in mothers (*n* = 5) in comparison to fathers (*n* = 1) and to both parents (*n* = 1). Among mothers, workplace inflexibility (S62), longer working hours (S16), nonstandard work schedules (S42), and work–family conflict (S9, S42) were associated with higher parental stress, whereas professional workplace support was associated with lower stress (S76). In the single fathers-only study, longer working hours were not associated with stress, but workplace inflexibility was associated with higher paternal stress (S62). One national survey conducted in China with both parents found that flexible working hours were associated with lower levels of parental stress (S41).

##### Child’s Characteristics

This sub-category (*n* = 22) includes child age, sex, and behavior/developmental factors associated with parental stress. Findings regarding the child’s age were mixed. Six studies (S9, S13, S19, S29, S33, S103) reported higher parental stress among parents of older children, whereas one study (S32) found higher stress among parents of younger children. Two studies (S19, S108) reported no significant association, while one study (S7) found no correlation for mothers but a positive association for fathers. Concerning children’s gender, one study reported higher stress among parents of boys (S7), but another found no association (S39).

Children’s emotional and behavioral problems were extensively studied (*n* = 14) with consistent results. In mothers, children’s difficult temperament (S24, S73, S86, S88, S89, S98), negative emotionality (S9, S64), problem behaviors (S33, S34), externalizing behaviors (S70), and negative affect (S6) were associated with higher maternal stress. In fathers, difficult temperament (S89) and negative emotionality (S71) were associated with higher stress. Infants’ difficult temperament and crying were related to higher stress for both parents (S103).

Child’s developmental or cognitive problems were less studied (*n* = 7), with generally consistent findings. Children’s developmental problems were associated with higher maternal stress (S33). Children’s sleep problems (S58) and feeding problems were related to higher stress for mothers and fathers (S103). However, children’s illness revealed mixed findings: two studies found a positive relation to stress (S29, S89), but another two studies found no association (S103, S108).

##### Sociocultural Factors

This sub-category was less studied (*n* = 12) and covers factors that operate beyond the personal or family level, such as cultural values, social policies, and global challenges. For instance, adherence to familism beliefs (i.e., cultural values emphasizing collectivism and strong family attachment) was associated with greater maternal stress in one study (S6), but it was associated with lower parental stress in a Mexican-origin sample (S52). In contrast, adherence to maternal essentialism (i.e., the belief that mothers are inherently more competent, important, and essential to a child's development than fathers) was associated with greater stress in mothers (S24) and fathers (S71).

Regarding social policies on parenting, one study found that taking maternal leave was associated with lower stress in mothers (S66), while another found that shared parental leave was related to lower stress in fathers, but not in mothers (S51). Among minority mothers, receiving a subsidy from the government for childcare was related to lower parental stress (S35).

Regarding global challenges, one study reported that factors such as war and global heating were associated with higher parental stress (S103). A key factor within the global challenges was the COVID-19 pandemic: lockdown during this period was associated with higher maternal stress in one study (S1), whereas another found no association (S30). Notably, restrictions on social contacts during the pandemic were associated with lower parental stress (S29).

### Stress Evolution and Comparison Between Mothers and Fathers

Although not part of our prespecified objectives, we also extracted, when available, (a) longitudinal data on parental stress trajectories during the child’s first three years (*n* = 17) and (b) differences between mothers’ and fathers’ stress levels (*n* = 13).

Regarding stress evolution, findings remain inconclusive. Eight studies reported increases in parental stress over time, from the first weeks postpartum to toddlerhood (S5, S39, S78) and within the toddler years (S25, S33, S46, S48, S54, S86). These were conducted primarily in Asia (S5, S48, S54) and the U.S. (S25, S46, S78, S86). Stable levels were found in some cases, from ages 2 to 3 years (S8, S9, S17). However, within the first six months after childbirth, one Portuguese (S3) and one Chinese (S106) study reported a decrease from the first weeks to 6 months postpartum. Studies conducted during COVID-19 found increases within the year following the initial lockdown (S1, S77, S88, S108).

Regarding the comparison between mothers' and fathers' stress, six studies revealed higher parental stress in mothers (S26, S44, S47, S55, S62, S82). In contrast, two studies found higher stress among fathers (S67, S89). In a pre–post COVID-19 comparison, mothers and fathers had similar stress levels before the pandemic, but fathers reported higher stress during the pandemic (S77). Finally, four studies found no significant differences between mothers and fathers (S37, S61, S72, S75).

## Discussion

The primary objective of this study was to provide a systematic review of the existing research on the antecedents of parental stress in early childhood. Relying on ecological parenting and parental stress models, this review aimed to: (1) characterize the included studies in terms of methodologies and populations used; (2) identify the variables examined as predictors of parental stress in the 0–3 years period; and (3) identify gaps in the literature to suggest directions for future studies. We moved forward from previous reviews by focusing specifically on parental stress in the general population and in children aged 0–3 years. Main contributions and gaps of the included studies are discussed below.

### Design, Measures, and Cultural Contexts of Parental Stress Research

We observed a predominance of longitudinal studies using self-report measures. The notable share of studies that adopted longitudinal approaches may reflect both the availability of cohort datasets (e.g., Early Head Start, FFCWS) and a broader methodological shift toward tracking change over time. Multiple waves help clarify determinants and outcomes, also offering a view of how stress unfolds. Regarding measures, the PSI (Abidin et al., 2006) was frequently used, which reflects its predominance in the parental stress field (Øygarden et al., 2022). However, some of PSI subscales (e.g., “Difficult Child”) include items less suited to the infant period, which may decrease sensitivity to early caregiving realities (Schappin et al., 2013). Despite the PSI predominance, some studies used the Parental Stress Scale (Berry & Jones, 1995), which provides a complementary perspective by including the bidirectional nature of the parent–child relationship (Louie et al., 2017).

Although most included studies relied on original data, a considerable number used secondary datasets (e.g., FFCWS). These datasets can enhance the representativeness of research, since they often focus on specific samples, such as vulnerable populations (Baldwin et al., 2022). However, some concerns emerge from their repeated use: some parental stress measures in these datasets were not validated scales, which may explain some of the inconsistent findings reported; additionally, repeated use of the same dataset across multiple studies may overemphasize certain findings (Tripathy, 2013). For example, this may help explain the mixed findings of education as a predictor of parental stress. Although 22 studies examined education, nine of them drew on overlapping datasets: studies S13, S29, and S83 used the same CoronaBy sample; studies S67 and S89 relied on the same Canadian sample; and studies S24, S62, S69, and S86 used data from the FFWCS.

Most studies were conducted in Western contexts, predominantly with White, married or cohabiting participants. However, an increasing number of studies have emerged from Asian and Middle Eastern countries, which differ from Western societies in terms of family functioning. Western cultures are generally individualistic, emphasizing equality within family relationships, with nuclear families being more common than extended ones (Hofstede, 1980). In contrast, the Asian and Middle Eastern cultures are characterized by a collectivist orientation, reflected in close family interdependence and more robust support systems (Hofstede, 1980). These cultural distinctions likely influence how parents experience stress, as well as which determinants become more salient in each context (Matias et al., 2023; Roskam et al., 2022). Specifically in our review, having a partner or higher income was unrelated to parental stress in European samples, but appeared more influential in other cultural contexts. Cultural differences were more evident in sociodemographic determinants and family context factors (e.g., social support) than in psychological or relational variables. This pattern may suggest that internal symptoms and within-family dynamics predict parental stress similarly across cultures, while sociodemographic conditions (e.g., partnership status, socioeconomic level, number of children) and available support systems differ in their influence.

Finally, as in much of the parenting literature, mothers were overrepresented, and fathers appeared less frequently (Cabrera et al., 2018; Volling & Palkovitz, 2021). Our review nonetheless identified more father-focused work than Fang et al.’s (2022) review, reflecting a growing attention to fatherhood. Even so, when both parents were included, they were often analyzed as a single group, which may hide gendered patterns and actor–partner dynamics. Designs that explicitly model interdependence (e.g., actor–partner interdependence models) may yield more nuanced insight into how stress is shaped within couples (Coutts et al., 2019; d’Orsi & Diniz, 2025; Westman, 2002).

### Risk and Protective Factors for Parental Stress in Early Childhood

Overall, the literature on parental stress has placed greater emphasis on risk factors than on protective ones, a pattern also observed by Fang et al. (2022). Within risk factors, parents’ psychological factors were the most commonly investigated in association with parental stress, with depressive symptoms playing an important role in increasing parental stress. This pattern is consistent with broader evidence on the mutual reinforcement of psychological symptoms and stress (Anding et al., 2016; Dennis et al., 2018). Parents experiencing depressive symptoms may struggle with emotion regulation, experience self-doubt, and reduce engagement with parenting responsibilities, which can make everyday caregiving feel more demanding and, in turn, heighten stress (Agostini et al., 2022; Alves et al., 2022; Vismara et al., 2016). Children’s emotional and behavioral problems were another highly studied risk domain: externalizing and internalizing difficulties likely require greater cognitive, emotional, and physical investment from parents, contributing to a sense of challenge and elevated stress (Alakortes et al., 2017; Szymańska & Aranowska, 2019; Van Der Pol et al., 2016).

Perinatal factors, though examined less often, also emerged as a risk factor for parental stress. Pregnancy, childbirth, and the postpartum period constitute a critical window during which parents reorganize their roles and learn to care for a new child (Bakermans‐Kranenburg et al., 2019). Mothers, in particular, navigate substantial physiological changes and demands associated with pregnancy, birth, the puerperium, and breastfeeding (Thurow, 2016). This phase is often characterized as stressful (Lévesque et al., 2020; Schoppe-Sullivan et al., 2021), and the studies identified in this review consistently suggest that adverse events and emotional distress heighten parental stress during this time. Another less studied risk factor was the COVID-19 pandemic and its associated changes. In some cases, circumstances during this period turned normally protective factors into risks, such as having to rely on daycare.

The protective factors studied more frequently were the mother-father relationship and social support. The quality of the couple relationship and coparenting were consistently found to buffer parental stress, suggesting that a cooperative partnership can mitigate individual stress during the high-demand early years (Schoppe-Sullivan et al., 2016). Social support within the family and informal networks similarly operated as a protective factor, aligning with the view that parenting is more manageable when embedded in strong support systems (Akaroğlu, 2023; Young et al., 2020). Such support may reduce stress by offering emotional connection, practical assistance, and advice (Knoester & Petts, 2017; Ranta et al., 2023).

Sociodemographic factors were widely examined but with mixed findings, in line with prior reviews (Fang et al., 2022; Philpott et al., 2017). Two context-dependent patterns seem to emerge. On the one hand, families facing structural disadvantage (e.g., low SES) seem to experience high parental stress due to financial constraints, limited time, and reduced access to supportive resources (Alen et al., 2020; Coba-Rodriguez & Lleras, 2022; Horne et al., 2022). On the other hand, more advantaged families (e.g., White, high SES) also seem to report elevated parental stress in some contexts, which can be explained by intensive parenting norms, information overload, and higher self-imposed and societal expectations (Meeussen & Van Laar, 2018; Mohr & Sonnentag, 2023; Venard et al., 2024). This pattern is illustrated in the findings by Parkes and colleagues (2015), which showed that mothers with either low or high levels of education reported more stress when compared with those with intermediate education. These mixed findings signal the need for further research but also invite a more optimistic interpretation: parental stress may be less dependent on sociodemographic factors (e.g., income and education) and more responsive to modifiable contexts and support (e.g., mental health, social support).

Although relatively few studies directly examine gender differences, the available evidence requires attention. According to the OECD Family Database (OECD, 2023), women across the globe tend to spend more time than men on care work as a primary activity, which likely contributes to greater burden and higher parental stress among mothers. Nevertheless, some studies in this review have reported higher stress among fathers than mothers. Fathers are also vulnerable to emotional difficulties in the perinatal period (Philpott et al., 2017; Widarsson et al., 2015), potentially reflecting lower emotional preparedness and less support from health professionals and informational resources (Knoester & Petts, 2017; Ranta et al., 2023), making them similarly susceptible to parental stress. Because research in this area has largely focused on mothers, it remains unclear whether risk and protective factors operate similarly for both parents; thus, more gender-sensitive research is needed.

In general, our findings align with Fang et al. (2022) in highlighting parents’ psychological symptoms, children’s problem behaviors, and social support as central predictors of parental stress. Our findings also extend prior work by underscoring the prominence of marital and coparenting quality and the importance of perinatal experiences, factors that are particularly salient in the early childhood phase.

### Strengths and Limitations

This review's main strength is the inclusion of a large number of studies, which provides a systematic review of the factors examined as antecedents of parental stress across multiple levels. However, this also represents a limitation, as the considerable heterogeneity in variables and methodologies made synthesis challenging, and findings related to specific variables should be interpreted with caution. Another strength is the inclusion of high-quality studies by following the criteria of Kmet and colleagues (2004).

Several limitations should be acknowledged. First, by including only peer-reviewed empirical articles published in the last 12 years, potentially relevant sources such as book chapters and grey literature were excluded, as they were not indexed in the databases searched. Second, in line with our focus on parental stress in typical family contexts, studies examining pathological processes or stress in high-risk contexts (e.g., incarcerated parents) were not included. Third, qualitative studies were also excluded due to our goal to identify predictors of parental stress, despite their potential to provide deeper insights into contradictory findings and less explored dimensions of parental stress. Although the review included studies in English, Portuguese, and Spanish, language restrictions may have introduced selection bias. Moreover, studies focused mostly on Western, married, and high-income mothers, which limits the generalizability of the findings to fathers and families from minority groups or low-resource settings. The limited representation of fathers further contributes to an overemphasis on maternal experiences and has implications not only for fathers’ mental health but also for the well-being of their partners and children (Bronfenbrenner & Morris, 1998; Cabrera et al., 2014, 2018).

Methodological limitations were also evident. Approximately one-third of the studies were cross-sectional, restricting the ability to infer causality; most associations should therefore be interpreted as correlational. In addition, all studies relied on self-report measures, which may introduce bias, particularly among fathers, who are known to underreport mental health difficulties (Bergin et al., 2013). Furthermore, despite excluding low-quality studies, a considerable number still failed to report key demographic characteristics, such as socioeconomic status, ethnicity, or parents’ employment status. Finally, in line with systematic review procedures, the magnitude of effect sizes was not assessed, limiting our ability to determine which determinants exert the strongest influence and to evaluate the consistency of findings across different contexts. Despite these limitations, this review represents an important step in mapping the determinants of parental stress through a systematic and ecological lens.

### Future Research and Practical Implications

Building on these insights, it is essential to consider how future research can address the identified gaps. First, there is a need to move beyond the overrepresentation of Western biological heterosexual families in literature to include more diverse samples (e.g., single-parent, ethnic minorities). This is essential to understand how parental stress operates across different socio-economic, cultural, and policy contexts (Masarik & Conger, 2017). Second, future studies should prioritize the inclusion of fathers and adopt dyadic designs to capture the interdependence between partners in shaping stress experiences, shifting from a maternal-centric perspective to a more holistic, family-oriented approach (Volling & Cabrera, 2025; Volling et al., 2019). Third, sociocultural factors received relatively limited attention, a finding consistent with Fang et al. (2022). Therefore, future research should further examine how cultural norms, parenting beliefs, and employment conditions contribute to parental stress. Given the rise of dual-earner households and evolving gender roles (Bianchi & Milkie, 2010; Diniz et al., 2025), future work could benefit from examining how job demands, flexibility, and workplace support shape parental stress, including spillover and work–family conflict pathways. At a broader level, societal norms, policy environments, and cultural expectations may play a significant role in shaping parental stress and should be addressed to capture differences across global contexts (Bronfenbrenner & Morris, 1998; Cabrera et al., 2014). Greater attention should also be given to the perinatal phase, as this period poses unique stressors related to breastfeeding, adapting to infant routines, sleep deprivation, and social isolation (Kline et al., 1991; Kuersten-Hogan & McHale, 2021). Finally, future research should increasingly employ longitudinal, multi-method approaches that combine self-report measures with observational or physiological data to capture the complexity of parental stress processes more accurately. We also recommend careful consideration of the parental stress measure, as some instruments may not be well-suited to the early childhood period (Øygarden et al., 2022).

Findings of this review also suggest important implications for clinical practice and public policy. Clinicians should screen parents for psychological symptoms in the perinatal phase, as these were consistently associated with higher levels of parental stress. This recommendation aligns with guidance from the American Academy of Pediatrics (American Academy of Pediatrics, 2022), which advocates for the identification of parental mental health difficulties in pediatric settings. Although screening for prenatal and postpartum depression is already recommended in several countries, its implementation remains inconsistent and not fully standardized across contexts (Australian Institute of Health & Welfare, 2024; Coker et al., 2024). We also suggest that clinicians provide targeted interventions to promote parental mental health during early childhood. Examples include the Mothers and Babies Program (Ahmed et al., 2023; Hamil et al., 2021), the ROSE program (Zlotnick et al., 2016), Be a Mom (Fonseca et al., 2019), and Supporting Father Involvement (Pruett et al., 2019), all of which have demonstrated effectiveness in reducing depressive symptoms and promoting parental well-being. Efforts to strengthen couples’ and coparenting relationships are essential, given their buffering effect on parental stress. Interventions should also include skills for problem-solving, mutual support, and shared parenting responsibilities, such as the Family Foundations (Feinberg et al., 2014) and the Baby Triple P (McPherson et al., 2022). At the community and policy level, building robust support networks for parents is crucial. Policies that promote family well-being, such as equitable and extended parental leave for both mothers and fathers, affordable and accessible childcare services, and family-friendly workplace practices, can help reduce structural stressors and improve family functioning.

Taken together, this review provides a comprehensive and ecologically grounded synthesis of the factors examined as antecedents of parental stress in early childhood. By identifying multilevel risk and protective factors, it underscores the importance of integrated approaches in research, practice, and policy to support parents and promote family well-being across diverse contexts.

## Electronic supplementary material

Below is the link to the electronic supplementary material.

Supplementary file1 (DOCX 42 KB)

## Data Availability

This study is a systematic review of previously published research. All data used in this study were extracted from the published articles included in the review, which are publicly available. The extracted datasets generated during the review are available from the corresponding author upon request.

## Appendix A: Included Studies and Study Identifiers

**Table:**

Study ID	Full citation
S1	Abufhele, A., Narea, M., & Telias, A. (2022). The Covid-19 Pandemic and Maternal Mental Health: A Longitudinal Study of Chilean and Foreign-Born Mothers. International Journal of Public Health, 67, 1604724. https://doi.org/10.3389/ijph.2022.1604724
S2	Alves, S., Fonseca, A., Canavarro, M. C., & Pereira, M. (2020). Intra‐couple similarity in dyadic coping and partners’ adjustment to the birth of a child. European Journal of Social Psychology, 50(1), 18–34. https://doi.org/10.1002/ejsp.2597
S3	Alves, S., Milek, A., Bodenmann, G., Fonseca, A., Canavarro, M. C., & Pereira, M. (2019). Romantic attachment, dyadic coping, and parental adjustment across the transition to parenthood. Personal Relationships, 26(2), 286–309. https://doi.org/10.1111/pere.12278
S4	Aubuchon-Endsley, N. L., Kennedy, T. S., Gilchrist, M., Thomas, D. G., & Grant, S. (2015). Relationships among Socioeconomic Status, Dietary Intake, and Stress in Breastfeeding Women. Journal of the Academy of Nutrition and Dietetics, 115(6), 939–946.e1. https://doi.org/10.1016/j.jand.2014.12.017
S5	Bahk, J., Yun, S.-C., Kim, Y., & Khang, Y.-H. (2015). Impact of unintended pregnancy on maternal mental health: A causal analysis using follow up data of the Panel Study on Korean Children (PSKC). BMC Pregnancy and Childbirth, 15(1), 85. https://doi.org/10.1186/s12884-015-0505-4
S6	Barnett, M. A., Mortensen, J. A., Tilley, E. H., & Gonzalez, H. (2013). Global and parenting-specific social support as protective factors for the well-being of Mexican American mothers of toddlers. Family Science, 4(1), 98–109. https://doi.org/10.1080/19424620.2013.807294
S7	Ben-Yaakov, O., & Ben-Ari, O. T. (2022). COVID-19-Related anxieties and parenting stress among first-time mothers and fathers in their first year of parenthood. Psychology & Health, 37(11), 1327–1341. https://doi.org/10.1080/08870446.2021.1942875
S8	Bergsund, H. B., Wentzel‐Larsen, T., & Jacobsen, H. (2020). [Parenting stress in long‐term foster carers: A longitudinal study]. Child & Family Social Work, 25(S1), 53–62. https://doi.org/10.1111/cfs.12713
S9	Berryhill, M. B., & Durtschi, J. A. (2017). Understanding Single Mothers’ Parenting Stress Trajectories. Marriage & Family Review, 53(3), 227–245. https://doi.org/10.1080/01494929.2016.1204406
S10	Blake, L., Jadva, V., & Golombok, S. (2014). Parent psychological adjustment, donor conception and disclosure: A follow-up over 10 years. Human Reproduction, 29(11), 2487–2496. https://doi.org/10.1093/humrep/deu231
S11	Boekhorst, M. G. B. M., Potharst, E. S., Beerthuizen, A., Hulsbosch, L. P., Bergink, V., Pop, V. J. M., & Nyklíček, I. (2020). Mindfulness during pregnancy and parental stress in mothers raising toddlers. Mindfulness, 11(7), 1747–1761. psyh. https://doi.org/10.1007/s12671-020-01392-9
S12	Borneskog, C., Lampic, C., Sydsjö, G., Bladh, M., & Skoog Svanberg, A. (2014). How do lesbian couples compare with heterosexual in vitro fertilization and spontaneously pregnant couples when it comes to parenting stress? Acta Paediatrica, 103(5), 537–545. https://doi.org/10.1111/apa.12568
S13	Buechel, C., Nehring, I., Seifert, C., Eber, S., Behrends, U., Mall, V., & Friedmann, A. (2022). A cross-sectional investigation of psychosocial stress factors in German families with children aged 0–3 years during the COVID-19 pandemic: Initial results of the CoronabaBY study. Child and Adolescent Psychiatry and Mental Health, 16(1). https://doi.org/10.1186/s13034-022-00464-z
S14	Camisasca, E., Di Blasio, P., Milani, L., & Miragoli, S. (2021). Postpartum depressive symptoms as a linking mechanism between maternal sleep and parenting stress: The conditional indirect effect by social support. Children’s Health Care, 50(1), 64–82. https://doi.org/10.1080/02739615.2020.1824675
S15	Canzi, E., Molgora, S., Fenaroli, V., Rosnati, R., Saita, E., & Ranieri, S. (2019). “Your stress is my stress”: A dyadic study on adoptive and biological first-time parents. Couple and Family Psychology: Research and Practice, 8(4), 197–207. https://doi.org/10.1037/cfp0000127
S16	Chatterji, P., Markowitz, S., & Brooks-Gunn, J. (2013). Effects of early maternal employment on maternal health and well-being. Journal of Population Economics, 26(1), 285–301. https://doi.org/10.1007/s00148-012-0437-5
S17	Chester, C. E., & Blandon, A. Y. (2016). Dual trajectories of maternal parenting stress and marital intimacy during toddlerhood. Personal Relationships, 23(2), 265–279. https://doi.org/10.1111/pere.12122
S18	Claridge, A. M., & Chaviano, C. L. (2013). Consideration of Abortion in Pregnancy: Demographic Characteristics, Mental Health, and Protective Factors. Women & Health, 53(8), 777–794. https://doi.org/10.1080/03630242.2013.831018
S19	Corner, G. W., Rasmussen, H. F., Khaled, M., Morris, A. R., Khoddam, H., Barbee, N., Herzig, S., Brasby, Y., Seibert, E., Sellery, P. E., Margolin, G., & Saxbe, D. (2023). The birth of a story: Childbirth experiences, meaning-making, and postpartum adjustment. Journal of Family Psychology, 37(5), 667–679. https://doi.org/10.1037/fam0001062
S20	Dai, Q., Lim, A. K., & Xu, Q. J. (2019). The relations between maternal mind-mindedness, parenting stress and obstetric history among Chinese mothers. Early Child Development and Care, 189(9), 1411–1424. https://doi.org/10.1080/03004430.2017.1385608
S21	De Cock, E. S. A., Henrichs, J., Klimstra, T. A., Janneke B. M. Maas, A., Vreeswijk, C. M. J. M., Meeus, W. H. J., & Van Bakel, H. J. A. (2017). Longitudinal Associations Between Parental Bonding, Parenting Stress, and Executive Functioning in Toddlerhood. Journal of Child and Family Studies, 26(6), 1723–1733. https://doi.org/10.1007/s10826-017-0679-7
S22	deMontigny, F., Gervais, C., Pierce, T., & Lavigne, G. (2020). Perceived Paternal Involvement, Relationship Satisfaction, Mothers’ Mental Health and Parenting Stress: A Multi-Sample Path Analysis. Frontiers in Psychiatry, 11, 578682. https://doi.org/10.3389/fpsyt.2020.578682
S23	Dollberg, D. G., Rozenfeld, T., & Kupfermincz, M. (2016). Early Parental Adaptation, Prenatal Distress, and High-Risk Pregnancy. Journal of Pediatric Psychology, 41(8), 915–929. https://doi.org/10.1093/jpepsy/jsw028
S24	Driver, N., & Shafeek Amin, N. (2019). Acculturation, Social Support, and Maternal Parenting Stress among U.S. Hispanic Mothers. Journal of Child and Family Studies, 28(5), 1359–1367. https://doi.org/10.1007/s10826-019-01351-6
S25	Fagan, J., & Lee, Y. (2014). Longitudinal Associations among Fathers’ Perception of Coparenting, Partner Relationship Quality, and Paternal Stress during Early Childhood. Family Process, 53(1), 80–96. https://doi.org/10.1111/famp.12055
S26	Faleschini, S., Aubuchon, O., Champeau, L., & Matte-Gagné, C. (2021). History of perinatal loss: A study of psychological outcomes in mothers and fathers after subsequent healthy birth. Journal of Affective Disorders, 280, 338–344. https://doi.org/10.1016/j.jad.2020.11.016
S27	Fomby, P. (2018). Motherhood in Complex Families. Journal of Family Issues, 39(1), 245–270. https://doi.org/10.1177/0192513X16637099
S28	Francis, L. M., Youssef, G. J., Greenwood, C. J., Enticott, P. G., Curtis, A., Graeme, L. G., Mansour, K. A., Olsson, C. A., Skouteris, H., Milgrom, J., Williams, J., Knight, T., & Macdonald, J. A. (2023). Father trait anger: Associations with father-infant bonding and subsequent parenting stress. Frontiers in Psychology, 14, 1114084. cmedm. https://doi.org/10.3389/fpsyg.2023.1114084
S29	Friedmann, A., Buechel, C., Seifert, C., Eber, S., Mall, V., & Nehring, I. (2023). Easing pandemic-related restrictions, easing psychosocial stress factors in families with infants and toddlers? Cross-sectional results of the three wave CoronabaBY study from Germany. Child and Adolescent Psychiatry and Mental Health, 17(1), 76. https://doi.org/10.1186/s13034-023-00618-7
S30	Galbally, M., Watson, S. J., Lewis, A. J., & Van IJzendoorn, M. H. (2022). Parenting stress, maternal depression and child mental health in a Melbourne cohort before and during the COVID‐19 pandemic. Journal of Paediatrics and Child Health, 58(11), 2051–2057. https://doi.org/10.1111/jpc.16155
S31	García-Blanco, A., Monferrer, A., Grimaldos, J., Hervás, D., Balanzá-Martínez, V., Diago, V., Vento, M., & Cháfer-Pericás, C. (2017). A preliminary study to assess the impact of maternal age on stress-related variables in healthy nulliparous women. Psychoneuroendocrinology, 78, 97–104. https://doi.org/10.1016/j.psyneuen.2017.01.018
S32	Gebhardt, A. J., Sydsjö, G., Skoog Svanberg, A., Indekeu, A., & Lampic, C. (2017). Parenting stress and its association with perceived agreement about the disclosure decision in parents following donor conception. Acta Obstetricia et Gynecologica Scandinavica, 96(8), 968–975. https://doi.org/10.1111/aogs.13157
S33	Goldberg, A. E., & Smith, J. Z. (2014). Predictors of parenting stress in lesbian, gay, and heterosexual adoptive parents during early parenthood. Journal of Family Psychology, 28(2), 125–137. https://doi.org/10.1037/a0036007
S34	Gonzalez, H., & Barnett, M. A. (2014). Romantic Partner and Biological Father Support: Associations with Maternal Distress in Low‐Income Mexican‐Origin Families. Family Relations, 63(3), 371–383. https://doi.org/10.1111/fare.12070
S35	Healy, O., & Dunifon, R. (2014). Child-Care Subsidies and Family Well-Being. Social Service Review, 88(3), 493–528. https://doi.org/10.1086/677741
S36	Henton, S., & Swanson, V. (2023). A mixed-methods analysis of the role of online social support to promote psychological wellbeing in new mothers. DIGITAL HEALTH, 9, 20552076221147433. https://doi.org/10.1177/20552076221147433
S37	Hildingsson, I., Haines, H., Johansson, M., Rubertsson, C., & Fenwick, J. (2014). Childbirth fear in Swedish fathers is associated with parental stress as well as poor physical and mental health. Midwifery, 30(2), 248–254. https://doi.org/10.1016/j.midw.2013.12.012
S38	Hildingsson, I., & Thomas, J. (2014). Parental stress in mothers and fathers one year after birth. Journal of Reproductive and Infant Psychology, 32(1), 41–56. https://doi.org/10.1080/02646838.2013.840882
S39	Holopainen, A., Verhage, M. L., & Oosterman, M. (2020). Childbirth experience associated with maternal and paternal stress during the first year, but not child attachment. Frontiers in Psychiatry, 11. psyh. https://doi.org/10.3389/fpsyt.2020.562394
S40	Hong, X., Zhu, W., & Luo, L. (2022). Non-parental Care Arrangements, Parenting Stress, and Demand for Infant–Toddler Care in China: Evidence From a National Survey. Frontiers in Psychology, 12, 822104. https://doi.org/10.3389/fpsyg.2021.822104
S41	Hong, X., Zhu, W., & Zhao, S. (2023). Type of Family Support for Infant and Toddler Care That Relieves Parenting Stress: Does the Number of Children Matter? Healthcare, 11(3), 421. https://doi.org/10.3390/healthcare11030421
S42	Hwang, W., & Jung, E. (2020). Unpartnered Mothers’ Work-Family Conflict and Parenting Stress: The Moderating Effects of Nonstandard Work Schedules. Journal of Family and Economic Issues, 41(1), 158–171. https://doi.org/10.1007/s10834-019-09647-x
S43	Imrie, S., Jadva, V., & Golombok, S. (2019). Psychological well-being of identity-release egg donation parents with infants. Human Reproduction, dez201. https://doi.org/10.1093/humrep/dez201
S44	Johansson, M., Svensson, I., Stenström, U., & Massoudi, P. (2017). Depressive symptoms and parental stress in mothers and fathers 25 months after birth. Journal of Child Health Care, 21(1), 65–73. https://doi.org/10.1177/1367493516679015
S45	Kang, S. K., Choi, H. J., & Chung, M. R. (2022). Coparenting and parenting stress of middle-class mothers during the first year: Bidirectional and unidirectional effects. Journal of Family Studies, 28(2), 551–568. https://doi.org/10.1080/13229400.2020.1744472
S46	Kanter, J. B., & Proulx, C. M. (2019). The longitudinal association between maternal parenting stress and spousal supportiveness. Journal of Family Psychology, 33(1), 121–131. https://doi.org/10.1037/fam0000478
S47	Kerstis, B., Nohlert, E., Öhrvik, J., & Widarsson, M. (2016). Association between depressive symptoms and parental stress among mothers and fathers in early parenthood: A Swedish cohort study. Upsala Journal of Medical Sciences, 121(1), 60–64. https://doi.org/10.3109/03009734.2016.1143540
S48	Kim, E. J., Cho, M. J., & Kim, M. J. (2021). Mothers’ Parenting Stress and Neighborhood Characteristics in Early Childhood (Ages 0–4). International Journal of Environmental Research and Public Health, 18(5). cmedm. https://doi.org/10.3390/ijerph18052648
S49	Knoester, C., & Petts, R. J. (2017). Fathers’ Parenting Stress After the Arrival of a New Child. Family Relations, 66(3), 367–382. https://doi.org/10.1111/fare.12263
S50	Liang, L. A., Berger, U., & Brand, C. (2019). Psychosocial factors associated with symptoms of depression, anxiety and stress among single mothers with young children: A population-based study. Journal of Affective Disorders, 242, 255–264. https://doi.org/10.1016/j.jad.2018.08.013
S51	Lidbeck, M., Bernhardsson, S., & Tjus, T. (2018). Division of parental leave and perceived parenting stress among mothers and fathers. Journal of Reproductive and Infant Psychology, 36(4), 406–420. https://doi.org/10.1080/02646838.2018.1468557
S52	Lindsey, E. W. (2022). Shared positive emotion during parent-toddler play and parent and child well-being in Mexican origin families. Infant Behavior and Development, 67, 101706. https://doi.org/10.1016/j.infbeh.2022.101706
S53	Maehara, K., Mori, E., Iwata, H., Sakajo, A., Aoki, K., & Morita, A. (2017). Postpartum maternal function and parenting stress: Comparison by feeding methods. International Journal of Nursing Practice, 23(S1), e12549. https://doi.org/10.1111/ijn.12549
S54	Matsumura, K., Hatakeyama, T., Yoshida, T., Tsuchida, A., Inadera, H., & The Japan Environment and Children’s Study (JECS) Group. (2023). Cesarean section and parenting stress: Results from the Japan Environment and Children’s Study. European Psychiatry, 66(1), e18. https://doi.org/10.1192/j.eurpsy.2023.5
S55	Matvienko-Sikar, K., Murphy, G., & Murphy, M. (2018). The role of prenatal, obstetric, and post-partum factors in the parenting stress of mothers and fathers of 9-month old infants. Journal of Psychosomatic Obstetrics & Gynecology, 39(1), 47–55. https://doi.org/10.1080/0167482X.2017.1286641
S56	Mazzeschi, C., Pazzagli, C., Radi, G., Raspa, V., & Buratta, L. (2015). Antecedents of maternal parenting stress: The role of attachment style, prenatal attachment, and dyadic adjustment in first-time mothers. Frontiers in Psychology, 6. https://doi.org/10.3389/fpsyg.2015.01443
S57	McCrory, C., & McNally, S. (2013). The Effect of Pregnancy Intention on Maternal Prenatal Behaviours and Parent and Child Health: Results of an Irish Cohort Study. Paediatric and Perinatal Epidemiology, 27(2), 208–215. https://doi.org/10.1111/ppe.12027
S58	Millikovsky-Ayalon, M., Atzaba-Poria, N., & Meiri, G. (2015). The role of the father in child sleep disturbance: child, parent, and parent–child relationship: Father’s Role in Child Sleep Disturbance. Infant Mental Health Journal, 36(1), 114–127. https://doi.org/10.1002/imhj.21491
S59	Moe, V., Von Soest, T., Fredriksen, E., Olafsen, K. S., & Smith, L. (2018). The Multiple Determinants of Maternal Parenting Stress 12 Months After Birth: The Contribution of Antenatal Attachment Style, Adverse Childhood Experiences, and Infant Temperament. Frontiers in Psychology, 9, 1987. https://doi.org/10.3389/fpsyg.2018.01987
S60	Molgora, S., Fenaroli, V., & Saita, E. (2020). The association between childbirth experience and mother’s parenting stress: The mediating role of anxiety and depressive symptoms. Women & Health, 60(3), 341–351. https://doi.org/10.1080/03630242.2019.1635563
S61	Nijssens, L., Bleys, D., Casalin, S., Vliegen, N., & Luyten, P. (2018). Parental Attachment Dimensions and Parenting Stress: The Mediating Role of Parental Reflective Functioning. Journal of Child and Family Studies, 27(6), 2025–2036. https://doi.org/10.1007/s10826-018-1029-0
S62	Nomaguchi, K., Brown, S., & Leyman, T. M. (2017). Fathers’ Participation in Parenting and Maternal Parenting Stress: Variation by Relationship Status. Journal of Family Issues, 38(8), 1132–1156. https://doi.org/10.1177/0192513X15623586
S63	Nomaguchi, K., & Johnson, W. (2016). Parenting Stress Among Low-Income and Working-Class Fathers: The Role of Employment. Journal of Family Issues, 37(11), 1535–1557. https://doi.org/10.1177/0192513X14560642
S64	Oddi, K. B., Murdock, K. W., Vadnais, S., Bridgett, D. J., & Gartstein, M. A. (2013). Maternal and Infant Temperament Characteristics as Contributors to Parenting Stress in the First Year Postpartum. Infant and Child Development, 22(6), 553–579. https://doi.org/10.1002/icd.1813
S65	Parkes, A., Sweeting, H., & Wight, D. (2015). Parenting stress and parent support among mothers with high and low education. Journal of Family Psychology, 29(6), 907–918. https://doi.org/10.1037/fam0000129
S66	Petts, R. J. (2018). Time Off After Childbirth and Mothers’ Risk of Depression, Parenting Stress, and Parenting Practices. Journal of Family Issues, 39(7), 1827–1854. https://doi.org/10.1177/0192513X17728984
S67	Rassart, C. A., Paradis, A., Bergeron, S., & Godbout, N. (2022). Cumulative childhood interpersonal trauma and parenting stress: The role of self-capacities disturbances among couples welcoming a newborn. Child Abuse & Neglect, 129, 105638. https://doi.org/10.1016/j.chiabu.2022.105638
S68	Riina, E. M. (2019). Neighborhood Qualities and Parenting Among Mothers With Young Children: Variation by Relationship Status. Journal of Family Issues, 40(15), 2076–2096. https://doi.org/10.1177/0192513X19849635
S69	Sampson, M., Villarreal, Y., & Padilla, Y. (2015). Association between Support and Maternal Stress at One Year Postpartum: Does Type Matter? Social Work Research, 39(1), 49–60. https://doi.org/10.1093/swr/svu031
S70	Schellinger, K. B., Murphy, L. E., Rajagopalan, S., Jones, T., Hudock, R. L., Graff, J. C., Palmer, F. B., & Tylavsky, F. A. (2020). Toddler Externalizing Behavior, Social Support, and Parenting Stress: Examining a Moderator Model. Family Relations, 69(4), 714–726. https://doi.org/10.1111/fare.12478
S71	Schoppe-Sullivan, S. J., Donithen, R. W., Lee, J., Simon, L. T., & Wang, J. (2021). The Best and Worst of Times: Predictors of New Fathers’ Parenting Satisfaction and Stress. Adversity and Resilience Science, 2(2), 71–83. https://doi.org/10.1007/s42844-021-00032-y
S72	Seah, C. K. F., & Morawska, A. (2016). When mum is stressed, is dad just as stressed? Predictors of paternal stress in the first six months of having a baby. Infant Mental Health Journal, 37(1), 45–55. https://doi.org/10.1002/imhj.21546
S73	Shai, D., Dollberg, D., & Szepsenwol, O. (2017). The importance of parental verbal and embodied mentalizing in shaping parental experiences of stress and coparenting. Infant Behavior and Development, 49, 87–96. https://doi.org/10.1016/j.infbeh.2017.08.003
S74	Skjothaug, T., Smith, L., Wentzel‐Larsen, T., & Moe, V. (2018). Does fathers’ prenatal mental health bear a relationship to parenting stress at 6 months? Infant Mental Health Journal, 39(5), 537–551. https://doi.org/10.1002/imhj.21739
S75	Stockdale, L., & Coyne, S. M. (2020). Parenting paused: Pathological video game use and parenting outcomes. Addictive Behaviors Reports, 11, 100244. https://doi.org/10.1016/j.abrep.2019.100244
S76	Swanson, V., & Hannula, L. (2022). Parenting stress in the early years – a survey of the impact of breastfeeding and social support for women in Finland and the UK. BMC Pregnancy and Childbirth, 22(1), 699. https://doi.org/10.1186/s12884-022-05010-5
S77	Taubman – Ben-Ari, O., Ben-Yaakov, O., & Chasson, M. (2021). Parenting stress among new parents before and during the COVID-19 pandemic. Child Abuse & Neglect, 117, 105080. https://doi.org/10.1016/j.chiabu.2021.105080
S78	Thomason, E., Volling, B. L., Flynn, H. A., McDonough, S. C., Marcus, S. M., Lopez, J. F., & Vazquez, D. M. (2014). Parenting stress and depressive symptoms in postpartum mothers: Bidirectional or unidirectional effects? Infant Behavior and Development, 37(3), 406–415. https://doi.org/10.1016/j.infbeh.2014.05.009
S79	Unternaehrer, E., Cost, K. T., Jonas, W., Dhir, S. K., Bouvette-Turcot, A.-A., Gaudreau, H., Dass, S. H., Lydon, J. E., Steiner, M., Szatmari, P., Meaney, M. J., & Fleming, A. S. (2019). Once and Again: History of Rearing Experiences and Psychosocial Parenting Resources at Six Months in Primiparous Mothers. Human Nature, 30(4), 448–476. a9h
S80	Van Rijn-van Gelderen, L., Bos, H. W. M., Jorgensen, T. D., Ellis-Davies, K., Winstanley, A., Golombok, S., Rubio, B., Gross, M., Vecho, O., & Lamb, M. E. (2018). Wellbeing of gay fathers with children born through surrogacy: A comparison with lesbian-mother families and heterosexual IVF parent families. Human Reproduction, 33(1), 101–108. https://doi.org/10.1093/humrep/dex339
S81	Vänskä, M., Punamäki, R.-L., Tolvanen, A., Lindblom, J., Flykt, M., Unkila-Kallio, L., Tulppala, M., & Tiitinen, A. (2017). Paternal mental health trajectory classes and early fathering experiences: Prospective study on a normative and formerly infertile sample. International Journal of Behavioral Development, 41(5), 570–580. https://doi.org/10.1177/0165025416654301
S82	Widarsson, M., Engström, G., Berglund, A., Tydén, T., & Lundberg, P. (2014). Parental stress and dyadic consensus in early parenthood among mothers and fathers in Sweden. Scandinavian Journal of Caring Sciences, 28(4), 689–699. https://doi.org/10.1111/scs.12096
S83	Widarsson, M., Engström, G., Rosenblad, A., Kerstis, B., Edlund, B., & Lundberg, P. (2013). Parental stress in early parenthood among mothers and fathers in Sweden. Scandinavian Journal of Caring Sciences, 27(4), 839–847. a9h
S84	Williams, D. T., & Parra, G. R. (2019). The longitudinal and bidirectional association between parenting stress and couples’ relationship quality. Personal Relationships, 26(4), 713–732. https://doi.org/10.1111/pere.12301
S85	Witcraft, S. M., Perry, M. M., Viana, A. G., Tull, M. T., & Dixon, L. J. (2024). A Preliminary Investigation of Prenatal Anxiety Sensitivity and Postpartum Distress. Journal of Midwifery & Women’s Health, 69(1), 58–63. https://doi.org/10.1111/jmwh.13529
S86	Xu, Y., Wang, X., Ahn, H., & Harrington, D. (2018). Predictors of non-U.S. born mothers’ parenting stress across early childhood in fragile families: A longitudinal analysis. Children and Youth Services Review, 89, 62–70. https://doi.org/10.1016/j.childyouth.2018.04.012
S87	Zhang, X., Middlemiss, W., Zhang, T., & Kelly, L. (2021). Mothers’ parenting stress in Chinese immigrant families: The role of fathers’ involvement and social support. Journal of Family Studies, 1–19. https://doi.org/10.1080/13229400.2021.1976250
S88	Aviles, A. I., Betar, S. K., Cline, S. M., Tian, Z., Jacobvitz, D. B., & Nicholson, J. S. (2024). Parenting young children during COVID-19: Parenting stress trajectories, parental mental health, and child problem behaviors. Journal of Family Psychology, 38(2), 296–308. https://doi.org/10.1037/fam0001181
S89	Bakhos, G., Villeneuve, É., Bélanger, C., Paradis, A., Brassard, A., Bergeron, S., & Godbout, N. (2024). Cumulative childhood interpersonal trauma and parental stress: The role of partner support. Journal of Social and Personal Relationships, 41(9), 2500–2521. https://doi.org/10.1177/02654075241246794
S90	Bentley, G., & Zamir, O. (2022). The Role of Maternal Self-efficacy in the Link Between Childhood Maltreatment and Maternal Stress During Transition to Motherhood. Journal of Interpersonal Violence, 37(21–22), NP19576–NP19598. https://doi.org/10.1177/08862605211042871
S91	Camisasca, E., Procaccia, R., Miragoli, S., Valtolina, G. G., & Di Blasio, P. (2017). Maternal mind-mindedness as a linking mechanism between childbirth-related posttraumatic stress symptoms and parenting stress. Health Care for Women International, 38(6), 593–612. https://doi.org/10.1080/07399332.2017.1296840
S92	Di Blasio, P., Camisasca, E., & Miragoli, S. (2018). Childbirth Related Post-traumatic Stress Symptoms and Maternal Sleep Difficulties: Associations With Parenting Stress. Frontiers in Psychology, 9, 2103. https://doi.org/10.3389/fpsyg.2018.02103
S93	Di Blasio, P., Camisasca, E., Miragoli, S., Ionio, C., & Milani, L. (2017). Does Maternal Parenting Stress Mediate the Association Between Postpartum PTS Symptoms and Children’s Internalizing and Externalizing Problems? A Longitudinal Perspective. Child & Youth Care Forum, 46(5), 685–701. https://doi.org/10.1007/s10566-017-9400-7
S94	Gong, Q. (2024). Transition to a new baby: Exploring the association between couple leisure, coparenting and parenting practices. Journal of Leisure Research, 1–16. https://doi.org/10.1080/00222216.2024.2374445
S95	Hsu, H.-C., Lee, S.-Y., Lai, C.-M., Tsai, W.-L., & Chiu, H.-T. (2018). Effects of Pediatric Anticipatory Guidance on Mothers of Young Children. Western Journal of Nursing Research, 40(3), 305–326. https://doi.org/10.1177/0193945916681292
S96	Huizink, A. C., Menting, B., De Moor, M. H. M., Verhage, M. L., Kunseler, F. C., Schuengel, C., & Oosterman, M. (2017). From prenatal anxiety to parenting stress: A longitudinal study. Archives of Women’s Mental Health, 20(5), 663–672. https://doi.org/10.1007/s00737-017-0746-5
S97	Kaneko, N., Nishijo, M., Agawa, K., Ishigaki, K., & Nishino, Y. (2024). The Effects of Neighborhood Trust and Support on Parenting Stress of Mothers With Young Children in Japan. Journal of Primary Care & Community Health, 15, 21501319241237056. https://doi.org/10.1177/21501319241237056
S98	Kit, A., Arima, K., Abe, Y., Mizukami, S., Tomita, Y., Hasegawa, M., Sou, Y., Nishimura, T., Ohnishi, M., & Aoyagi, K. (2022). Association between Mothers’ Attachment Styles and Parenting Stress among Japanese Mothers with Toddlers. Psychiatry International, 3(2), 122–130. https://doi.org/10.3390/psychiatryint3020010
S99	Mitev, K., Frewin, K. L., Augustinova, M., Niedenthal, P. M., Rychlowska, M., & Vanderwert, R. E. (2024). The who, when, and why of pacifier use. Pediatric Research. https://doi.org/10.1038/s41390-024-03540-6
S100	Nakajima, M. (2024). The moderating effect of maternal childhood maltreatment on the association between social support and parenting outcomes. Psychiatry Research Communications, 4(4), 100198. https://doi.org/10.1016/j.psycom.2024.100198
S101	Nam, Y., Wikoff, N., & Sherraden, M. (2015). Racial and Ethnic Differences in Parenting Stress: Evidence from a Statewide Sample of New Mothers. Journal of Child and Family Studies, 24(2), 278–288. https://doi.org/10.1007/s10826-013-9833-z
S102	Pazzagli, C., Buratta, L., Coletti, E., & Mazzeschi, C. (2023). Mother-to-infant bonding mediates the effects of depressive and anxious postpartum symptoms on parenting stress. Journal of Psychosomatic Obstetrics & Gynecology, 44(1), 2264487. https://doi.org/10.1080/0167482X.2023.2264487
S103	Richter, K., Buechel, C., Augustin, M., Friedmann, A., Mall, V., & Nehring, I. (2025). Psychosocial stress in families of young children after the pandemic: No time to rest. Child and Adolescent Psychiatry and Mental Health, 19(1), 50. https://doi.org/10.1186/s13034-025-00905-5
S104	Sacchi, C., Girardi, P., Buri, A., De Carli, P., & Simonelli, A. (2024). The perinatal health secondary to pandemic: Association between women’s delivery concerns and infant’s behavioral problems. Journal of Reproductive and Infant Psychology, 1–16. https://doi.org/10.1080/02646838.2024.2330662
S105	Shreffler, K. M., Dressler, C. M., Ciciolla, L., Wetherill, M. S., & Croff, J. M. (2024). Maternal periconception food insecurity and postpartum parenting stress and bonding outcomes. Frontiers in Nutrition, 11, 1275380. https://doi.org/10.3389/fnut.2024.1275380
S106	Wang, Y., Gu, J., Gao, Y., Lu, Y., Zhang, F., & Xu, X. (2023). Postpartum stress in the first 6 months after delivery: A longitudinal study in Nantong, China. BMJ Open, 13(10), e073796. https://doi.org/10.1136/bmjopen-2023-073796
S107	Zhang, S., Aquino, G. A., Tian, Z., Hazen, N., & Jacobvitz, D. (2023). Mothers’ resilience and potential for disrupted parenting in COVID-19: The protective effect of cognitive reappraisal. Journal of Family Psychology, 37(5), 603–613. https://doi.org/10.1037/fam0001103
S108	Buechel, C., Friedmann, A., Eber, S., Behrends, U., Mall, V., & Nehring, I. (2024). The change of psychosocial stress factors in families with infants and toddlers during the COVID-19 pandemic. A longitudinal perspective on the CoronabaBY study from Germany. Frontiers in Pediatrics, 12, 1354089. https://doi.org/10.3389/fped.2024.1354089
