# Supplementary material for: What Makes Parents of Young Children Stressed? A Systematic Review of Quantitative Studies
Source: Clin Child Fam Psychol Rev. 2026 Jun 2;29(3):571–93. doi: 10.1007/s10567-026-00573-7 (PMC13562140; doi:10.1007/s10567-026-00573-7)
Supplement: Supplementary file 1 [file 10567_2026_573_MOESM1_ESM.docx]

# Supplementary material

# What makes parents of young children stressed? A systematic review of quantitative studies

**Dora d’Orsi** (0000-0003-0537-1815) - William James Center for Research, Ispa—Instituto Universitário | Rua Jardim do Tabaco, 34, 1149-041 Lisbon, Portugal

**Carolina Garraio** (0000-0001-6368-8306) - Center for Psychology at University of Porto, Faculty of Psychology and Education Sciences, University of Porto | Rua Alfredo Allen, 4200-135 Porto, Portugal

**Rita Santos** - Ispa—Instituto Universitário | Rua Jardim do Tabaco, 34, 1149-041 Lisbon, Portugal

**Manuela Veríssimo** (0000-0003-4356-5696) - William James Center for Research, Ispa—Instituto Universitário | Rua Jardim do Tabaco, 34, 1149-041 Lisbon, Portugal

**Marisa Matias** (0000-0002-5755-5096) - Center for Psychology at University of Porto, Faculty of Psychology and Education Sciences, University of Porto | Rua Alfredo Allen, 4200-135 Porto, Portugal

**Eva Diniz** (0000-0003-4544-4839) - William James Center for Research, Ispa—Instituto Universitário | Rua Jardim do Tabaco, 34, 1149-041 Lisbon, Portugal

Corresponding author’s contact email address: [dora.duarte@ispa.pt](mailto:dora.duarte@ispa.pt)

| **Table 3.** *Personal determinants of parental stress according to sample type and direction of associations.* | | | | | | | | | |
| --- | --- | --- | --- | --- | --- | --- | --- | --- | --- |
|  | **Mothers** | | | **Fathers** | | | **Both parents** | | |
|  | **Negative (-)** | **Null (0)** | **Positive (+)** | **(-)** | **(0)** | **(+)** | **(-)** | **(0)** | **(+)** |
| **Personal history** |  |  |  |  |  |  |  |  |  |
| Adverse childhood experiences |  |  | #3, #50, #67, #79, #89, #90, #100 |  | #74 | #67, #89 |  |  |  |
| Attachment factors |  |  | #56, #59, #61, #98 |  |  | #61 |  |  |  |
| Parenting experience |  |  | #83 |  | #83 |  |  |  |  |
| Role model | #83, #79 |  |  | #83 |  |  |  |  |  |
| **Sociodemographic aspects** |  |  |  |  |  |  |  |  |  |
| Age |  | #39, #89, #90 | #1, #31, #67, #86, #106 | #89 | #39, #67, #74 |  |  | #103, #108 |  |
| Education | #24, #50, #62, #65, #69, #83, #86, #95 | #90 | #39, #67, #76, #89, #101, #106 | #49, #62 | #74, #83 | #39, #67, #89 |  | #103, #108 | #13, #29, #77 |
| Income | #6, #20, #24, #64, #76, #95, #98, #105 | #46, #72 | #88, #90 |  | #74 | #49, #72 | #13, #29 | #103, #108 |  |
| Having a partner | #1, #50, #63, #95, #107 | #27, #39, #62 |  | #62 | #39 |  |  | #103, #108 |  |
| Being employed | #16, #50, #55, #62 |  |  | #49, #62 | #55 |  |  |  |  |
| Migrant background |  | #62 | #1, #24, #39, #50, #76, #86, #95, #101 | #49 |  | #39, #62 |  |  |  |
| **General health** |  |  |  |  |  |  |  |  |  |
| Sleep difficulties |  |  | #14, #55, #93 |  |  |  |  |  |  |
| Physical health | #4, #55, #106 |  |  | #55 |  |  | #7, #77 |  |  |
| **Psychological aspects** |  |  |  |  |  |  |  |  |  |
| Internalizing symptoms (e.g., depressive symptoms) |  | #72, #104 | #9, #14, #17, #23, #24, #33, #44, #47, #60, #62, #64, #67, #75, #78, #86, #88, #96, #101, #102, #104, #106, #107 |  | #74 | #23, #28, #44, #47, #71, #72, #75, #82 |  |  | #29, #108 |
| Cognitive factors (e.g., self-efficacy, resilience) | #11, #47, #64, #76, #83, #91, #107 | #19, #67, #73, #87 | #67 | #19, #47, #49, #71, #72, #83 | #19, #67 |  | #61, #77 |  |  |
| **Perinatal factors** |  |  |  |  |  |  |  |  |  |
| Pregnancy intention | #57 |  | #18 |  |  |  | #5 |  |  |
| Pregnancy risk |  |  |  |  |  |  | #23 |  | #26 |
| Prenatal emotional experiences | #37 | #104 | #23, #85, #96 | #37 |  | #38, #74 |  |  |  |
| Mothers’ birth experience |  |  | #20, #39, #60, #91, #92, #93 |  |  | #39 |  |  | #77 |
| Type of labor |  |  | #54, #55 |  | #55 |  |  |  |  |
| Breastfeeding factors | #53 | #53 | #76 |  |  |  |  |  |  |
| Pacifier users | #99 |  |  |  |  |  |  |  |  |

| **Table 4.** *Relational determinants of parental stress according to sample type and direction of associations.* | | | | | | | | | | | | | | | | | | | | |  |  |
| --- | --- | --- | --- | --- | --- | --- | --- | --- | --- | --- | --- | --- | --- | --- | --- | --- | --- | --- | --- | --- | --- | --- |
|  | **Mothers** | | | | | | **Fathers** | | | | | | | | | **Both parents** | | | | | | |
|  | **Negative (-)** | | **Null (0)** | | **Positive (+)** | | | **(-)** | | **(0)** | | **(+)** | | **(-)** | | | **(0)** | | **(+)** | |  |  |
| **Mother-Father relationship** | |  | |  | |  | | |  | |  | |  | |  | | |  | |  | |  |
| Marital relationship quality | | #2, #17, #19, #22, #27, #46, #55, #56, #94 | | #3, #56, #84, #94 | | #56 | | | #2, #19, #25, #55, #107 | | #3, #19, #84, #94 | | #74 | | #32, #77, #83 | | |  | |  | |  |
| Coparenting quality | | #22, #24, #45, #63, #69, #73, #94 | |  | |  | | | #49, #94 | |  | | #25 | |  | | | #32 | | #29 | |  |
| **Cross-over effects** | |  | |  | |  | | |  | |  | |  | |  | | |  | |  | |  |
| Partner psychological factors | | #2, #3, #19 | | #15, #19, #67 | | #47, #67 | | | #2 | | #3, #19 | | #15, #19, #47, #67 | |  | | |  | |  | |  |
| Partner involvement with the child | | #22, #58, #63, #87 | | #86 | | #27 | | | #49 | |  | |  | |  | | |  | |  | |  |
| **Parent-Child relationship** | |  | |  | |  | | |  | |  | |  | |  | | |  | |  | |  |
| Quality of parent-child relationship | | #21, #52, #72, | | #21 | |  | | | #21, #28, #72 | | #21, #72 | |  | |  | | |  | |  | |  |
| Involvement with the child | | #9, #86 | |  | | #62 | | | #49, #58 | |  | |  | |  | | |  | |  | |  |

| **Table 5.** *Contextual* *determinants of parental stress according to sample type and direction of associations.* | | | | | | | | | |
| --- | --- | --- | --- | --- | --- | --- | --- | --- | --- |
|  | **Mothers** | | | **Fathers** | | | **Both parents** | | |
|  | **Negative (-)** | **Null (0)** | **Positive (+)** | **(-)** | **(0)** | **(+)** | **(-)** | **(0)** | **(+)** |
| **Family context** |  |  |  |  |  |  |  |  |  |
| Number of children | #50 | #107 | #1, #62, #98 | #37, #74 | #62 | #49 |  | #13 | #29, #41, #103 |
| Reproductive pathways |  |  |  |  |  |  | #12 | #10, #15, #43, #80 | #8 |
| Support from friends and family | #6, #14, #27, #33, #50, #65, #69, #70, #76, #86, #87, #89, #98, #100, #101, #107 | #34, #104 |  | #89 |  |  | #41 |  |  |
| External support | #48, #68, #95, #97 | #36 |  |  |  |  | #41 | #40 | #29, #103 |
| Work-related factors | #76 |  | #9, #16, #42, #62 |  | #62 | #62 |  |  |  |
| **Child’s characteristics** |  |  |  |  |  |  |  |  |  |
| Child's age |  | #7 | #9, #19, #33 |  | #19 | #7 | #32 | #108 | #13, #29, #103 |
| Child’s gender (ref, boy) |  | #39 | #7 |  | #39 | #7 |  |  |  |
| Child's emotional/behavioral problems |  |  | #6, #9, #24, #33, #34, #64, #70, #73, #86, #88, #89, #98 |  |  | #71, #89 |  |  | #103 |
| Child's developmental/cognitive problems |  |  | #33, #58 |  |  | #58, #89 |  | #103, #108 | #29, #103 |
| **Sociocultural factors** |  |  |  |  |  |  |  |  |  |
| Societal challenges |  |  |  |  |  |  |  |  | #103 |
| Social policies on parenting | #66 | #35, #51 |  | #51 |  |  |  |  |  |
| Covid-19 lockdown |  | #30 | #1 |  |  |  | #29 |  | #77 |
| Adherence to familism beliefs | #52 |  | #6 | #52 |  |  |  |  |  |
| Adherence to maternal essentialism |  |  | #24 |  |  | #71 |  |  |  |

| **Table 6.** *Standard Quality Assessment Criteria for Evaluating Primary Research Papers from various fields (QualSyst) for quantitative studies (Kmet et al., 2004)* | |
| --- | --- |
| Question or objective sufficiently described? | **Yes**: Is easily identified in the introductory section (or first paragraph of methods section). Specifically (where applicable, depending on study design) all of the following: purpose, subjects/target population, and the specific intervention(s) /association(s)/descriptive parameter(s) under investigation. A study purpose that only becomes apparent after studying other parts of the paper is not considered sufficiently described.  **Partial**: Vaguely/incompletely reported (e.g. “describe the effect of” or “examine the role of” or “assess opinion on many issues” or “explore the general attitudes”...); or some information has to be gathered from parts of the paper  other than the introduction/background/objective section.  **No**: Question or objective is not reported, or is incomprehensible. |
| Design evident and appropriate to answer study question? (If the study question is not given, infer from the conclusions). | **Yes**: Design is easily identified and is appropriate to address the study question /objective.  **Partial**: Design and /or study question not clearly identified, but gross inappropriateness is not evident; or design is easily identified but only partially addresses the study question.  **No**: Design used does not answer study question (e.g., a comparison group is required to answer the study question, but none was used); or design cannot be identified. |
| Method of subject selection (and comparison group selection, if applicable) or source of information/input variables (e.g., for decision analysis) is described and appropriate. | **Yes**: Described and appropriate. Selection strategy designed (i.e., consider sampling frame and strategy) to obtain an unbiased sample of the relevant target population or the entire target population of interest (e.g., consecutive patients  for clinical trials, population-based random sample for case-control studies or surveys). Where applicable, inclusion/exclusion criteria are described and defined (e.g., “cancer” -- ICD code or equivalent should be provided). Studies of volunteers: methods and setting of recruitment reported. Surveys: sampling frame/ strategy clearly described and appropriate.  **Partial**: Selection methods (and inclusion/exclusion criteria, where applicable) are not completely described, but no obvious inappropriateness. Or selection strategy is not ideal (i.e., likely introduced bias) but did not likely seriously  distort the results (e.g., telephone survey sampled from listed phone numbers only; hospital based case-control study identified all cases admitted during the study period, but recruited controls admitted during the day/evening only). Any  study describing participants only as “volunteers” or “healthy volunteers”. Surveys: target population mentioned but sampling strategy unclear.  **No**: No information provided. Or obviously inappropriate selection procedures (e.g., inappropriate comparison group if intervention in women is compared to intervention in men). Or presence of selection bias which likely seriously  distorted the results (e.g., obvious selection on “exposure” in a case-control study). |
| Subject (and comparison group, if applicable) characteristics or input variables/information (e.g., for decision analyses) sufficiently described? | **Yes**: Sufficient relevant baseline/demographic information clearly characterizing the participants is provided (or reference to previously published baseline data is provided). Where applicable, reproducible criteria used to describe/categorize the participants are clearly defined (e.g., ever-smokers, depression scores, systolic blood pressure > 140). If “healthy volunteers” are used, age and sex must be reported (at minimum). Decision analyses: baseline estimates for input variables are clearly specified.  **Partial**: Poorly defined criteria (e.g. “hypertension”, “healthy volunteers”, “smoking”). Or incomplete relevant baseline / demographic information (e.g., information on likely confounders not reported). Decision analyses: incomplete reporting of baseline estimates for input variables.  **No**: No baseline / demographic information provided. Decision analyses: baseline estimates of input variables not given. |
| Outcome and (if applicable) exposure measure(s) well defined and robust to measurement / misclassification bias? Means of assessment reported? | **Yes**: Defined (or reference to complete definitions is provided) and measured according to reproducible, “objective” criteria (e.g., death, test completion – yes/no, clinical scores). Little or minimal potential for measurement /  misclassification errors. Surveys: clear description (or reference to clear description) of questionnaire/interview content and response options. Decision analyses: sources of uncertainty are defined for all input variables.  **Partial**: Definition of measures leaves room for subjectivity, or not sure (i.e., not reported in detail, but probably acceptable). Or precise definition(s) are missing, but no evidence or problems in the paper that would lead one to  assume major problems. Or instrument/mode of assessment(s) not reported. Or misclassification errors may have occurred, but they did not likely seriously distort the results (e.g., slight difficulty with recall of long-ago events; exposure is measured only at baseline in a long cohort study). Surveys: description of questionnaire/interview content incomplete; response options unclear. Decision analyses: sources of uncertainty are defined only for some input variables.  **No**: Measures not defined, or are inconsistent throughout the paper. Or measures employ only ill-defined, subjective assessments, e.g. “anxiety” or “pain.” Or obvious misclassification errors/measurement bias likely seriously distorted  the results (e.g., a prospective cohort relies on self-reported outcomes among the “unexposed” but requires clinical assessment of the “exposed”). Surveys: no description of questionnaire/interview content or response options. Decision analyses: sources of uncertainty are not defined for input variables. |
| Sample size appropriate? | **Yes**: Seems reasonable with respect to the outcome under study and the study design. When statistically significant results are achieved for major outcomes, appropriate sample size can usually be assumed, unless large standard errors  (SE > ½ effect size) and/or problems with multiple testing are evident. Decision analyses: size of modeled cohort / number of iterations specified and justified.  **Partial**: Insufficient data to assess sample size (e.g., sample seems “small” and there is no mention of power/sample size/effect size of interest and/or variance estimates aren’t provided). Or some statistically significant results with standard errors > ½ effect size (i.e., imprecise results). Or some statistically significant results in the absence of variance estimates. Decision analyses: incomplete description or justification of size of modeled cohort/number of iterations.  **No**: Obviously inadequate (e.g., statistically non-significant results and standard errors > ½ effect size; or standard deviations > _ of effect size; or statistically non-significant results with no variance estimates and obviously inadequate sample size). Decision analyses: size of modeled cohort / number of iterations not specified. |
| Analysis described and appropriate? | **Yes**: Analytic methods are described (e.g. “chi square”/ “t-tests”/“Kaplan-Meier with log rank tests”, etc.) and appropriate.  Partial: Analytic methods are not reported and have to be guessed at, but are probably appropriate. Or minor flaws or some tests appropriate, some not (e.g., parametric tests used, but unsure whether appropriate; control group exists but  is not used for statistical analysis). Or multiple testing problems not addressed.  **No**: Analysis methods not described and cannot be determined. Or obviously inappropriate analysis methods (e.g., chi-square tests for continuous data, SE given where normality is highly unlikely, etc.). Or a study with a descriptive goal  / objective is over-analyzed. |
| Some estimate of variance (e.g., confidence intervals, standard errors) is reported for the main results/outcomes (i.e., those directly addressing the study question/objective upon which the conclusions are based)? | **Yes**: Appropriate variances estimate(s) is/are provided (e.g., range, distribution, confidence intervals, etc.). Decision analyses: sensitivity analysis includes all variables in the model.  **Partial**: Undefined “+/-“ expressions. Or no specific data given, but insufficient power acknowledged as a problem. Or variance estimates not provided for all main results/outcomes. Or inappropriate variance estimates (e.g., a study  examining change over time provides a variance around the parameter of interest at “time 1” or “time 2”, but does not provide an estimate of the variance around the difference). Decision analyses: sensitivity analysis is limited,  including only some variables in the model.  **No**: No information regarding uncertainty of the estimates. Decision analyses: No sensitivity analysis. |
| Controlled for confounding? | **Yes**: Randomized study, with comparability of baseline characteristics reported (or non-comparability controlled for in the analysis). Or appropriate control at the design or analysis stage (e.g., matching, subgroup analysis, multivariate  models, etc). Decision analyses: dependencies between variables fully accounted for (e.g., joint variables are considered).  **Partial**: Incomplete control of confounding. Or control of confounding reportedly done but not completely described. Or randomized study without report of comparability of baseline characteristics. Or confounding not considered, but  not likely to have seriously distorted the results. Decision analyses: incomplete consideration of dependencies between variables.  **No**: Confounding not considered, and may have seriously distorted the results. Decision analyses: dependencies between variables not considered. |
| Results reported in sufficient detail? | **Yes**: Results include major outcomes and all mentioned secondary outcomes.  **Partial**: Quantitative results reported only for some outcomes. Or difficult to assess as study question/objective not fully described (and is not made clear in the methods section), but results seem appropriate.  **No**: Quantitative results are reported for a subsample only, or “n” changes continually across the denominator (e.g., reported proportions do not account for the entire study sample, but are reported only for those with complete data  -- i.e., the category of “unknown” is not used where needed). Or results for some major or mentioned secondary outcomes are only qualitatively reported when quantitative reporting would have been possible (e.g., results include  vague comments such as “more likely” without quantitative report of actual numbers). |
| Do the results support the conclusions? | **Yes**: All the conclusions are supported by the data (even if analysis was inappropriate). Conclusions are based on all results relevant to the study question, negative as well as positive ones (e.g., they aren’t based on the sole  significant finding while ignoring the negative results). Part of the conclusions may expand beyond the results, if made in addition to rather than instead of those strictly supported by data, and if including indicators of their interpretative  nature (e.g., “suggesting,” “possibly”).  **Partial**: Some of the major conclusions are supported by the data, some are not. Or speculative interpretations are not indicated as such. Or low (or unreported) response rates call into question the validity of generalizing the results to the  target population of interest (i.e., the population defined by the sampling frame/strategy).  **No**: None or a very small minority of the major conclusions are supported by the data. Or negative findings clearly due to low power are reported as definitive evidence against the alternative hypothesis. Or conclusions are missing. Or  extremely low response rates invalidate generalizing the results to the target population of interest (i.e., the population defined by the sampling frame/strategy). |
